# Menin orchestrates hepatic glucose and fatty acid uptake via deploying the cellular translocation of SIRT1 and PPARγ

**DOI:** 10.1186/s13578-023-01119-y

**Published:** 2023-09-22

**Authors:** Tingjun Liu, Ranran Li, Lili Sun, Zhongjin Xu, Shengxuan Wang, Jingxuan Zhou, Xuanning Wu, Kerong Shi

**Affiliations:** 1 Laboratory of Animal Stem Cell and Reprogramming, College of Animal Science and Technology, Shandong Agricultural University, No. 61 Daizong Street, Taian, 271018 Shandong People’s Republic of China; 2 Key Laboratory of Animal Bioengineering and Disease Prevention of Shandong Province, Taian, 271018 Shandong People’s Republic of China

**Keywords:** Menin, Hepatocytes, PPARγ, SIRT1, NAFLD, Metabolism homeostatsis

## Abstract

**Background:**

Menin is a scaffold protein encoded by the *Men1* gene, which interacts with various transcriptional proteins to activate or repress cellular processes and is a key mediator in multiple organs. Both liver-specific and hepatocyte-specific Menin deficiency promotes high-fat diet-induced liver steatosis in mice, as well as insulin resistance and type 2 diabetic phenotype. The potential link between Menin and hepatic metabolism homeostasis may provide new insights into the mechanism of fatty liver disease.

**Results:**

Disturbance of hepatic Menin expression impacts metabolic pathways associated with non-alcoholic fatty liver disease (NAFLD), including the FoxO signaling pathway, which is similar to that observed in both oleic acid-induced fatty hepatocytes model and biopsied fatty liver tissues, but with elevated hepatic Menin expression and inhibited FABP1. Higher levels of Menin facilitate glucose uptake while restraining fatty acid uptake. Menin targets the expression of FABP3/4/5 and also CD36 or GK, PCK by binding to their promoter regions, while recruiting and deploying the cellular localization of PPARγ and SIRT1 in the nucleus and cytoplasm. Accordingly, Menin binds to PPARγ and/or FoxO1 in hepatocytes, and orchestrates hepatic glucose and fatty acid uptake by recruiting SIRT1.

**Conclusion:**

Menin plays an orchestration role as a transcriptional activator and/or repressor to target downstream gene expression levels involved in hepatic energy uptake by interacting with the cellular energy sensor SIRT1, PPARγ, and/or FoxO1 and deploying their translocations between the cytoplasm and nucleus, thereby maintaining metabolic homeostasis. These findings provide more evidence suggesting Menin could be targeted for the treatment of hepatic steatosis, NAFLD or metabolic dysfunction-associated fatty liver disease (MAFLD), and even other hepatic diseases.

**Graphical Abstract:**

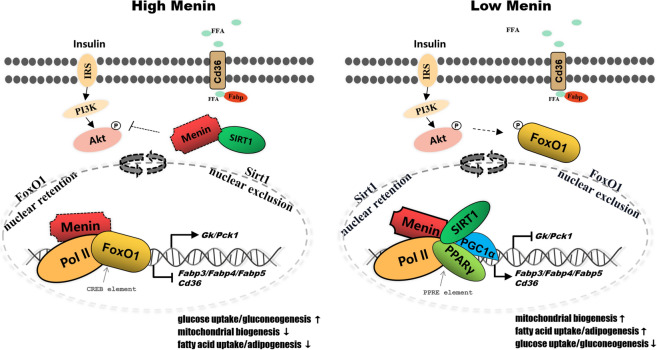

**Supplementary Information:**

The online version contains supplementary material available at 10.1186/s13578-023-01119-y.

## Highlights

- Higher Menin facilitates hepatic glucose uptake, but restrains hepatic fatty acid uptake. Conversely, lower Menin has the opposite effect.
- Menin interacts with PPARγ and/or FoxO1, as well as SIRT1, thereby targeting downstream gene expression.
- Through its recruitment of various proteins, Menin facilitates their translocations within the nucleus and cytoplasm.
- Menin serves as a key regulator of hepatic metabolism homeostasis.
- Menin expression is elevated in both induced fatty hepatocyte model and fatty liver tissues.

**Supplementary Information:**

The online version contains supplementary material available at 10.1186/s13578-023-01119-y.

## Introduction

Menin, a product of the tumor suppressor gene *Men1*, plays a crucial role in cellular growth and development processes [46], as confirmed by constructing the Men1 knockout mouse model (Men1^−/−^). Homozygous mutant mouse embryos died at day 11.5 ~ 12.5, accompanied by multiple organ defects [5, 6, 12]. Menin is abundantly expressed in multiple organs, especially tissues closely related to metabolism, participating in regulating metabolic activities [40, 49, 54]. As a scaffold protein, Menin recruits different factors and shuttles back and forth between the nucleus and cytoplasm [7, 47]. In the nucleus, Menin participates in transcriptional regulation, DNA replication, and DNA repair [23, 31]. As a transcription regulator, Menin participates in both suppression and activation of transcription. For instance, Menin acts as a co-repressor of transcription by binding to histone deacetylation complex HDAC or NAD-dependent deacetylase SIRT1 [8, 28], DNA methylation transferase [13, 54], and special transcriptional factors such as LXRα [10]. Conversely, Menin also plays its role as an activator of transcription by binding to special transcriptional factors such as PPARγ [14]. Menin, as a tumor suppressor, has established roles in epigenetic regulation and tumor suppression in multiple cancers such as prostate cancer, breast cancer, liver cancer, and lung cancer. Nowadays, the Menin-MLL (mixed lineage leukemia) interaction is regarded as a potential therapeutic strategy for leukemia and cancers. Many novel, potent, selective small molecules focused on the interaction have been discovered [30, 35, 44, 49].

In addition, Menin located in the cytoplasm can respond to glucose and/or insulin in multiple cell types, such as pancreatic β cells [57], mammary gland epithelial cells [33], and hepatocytes [48]. Menin's ability to sense the cellular environment provides a crucial prerequisite for its regulation of cellular and tissue metabolic homeostasis. Intriguing recent data suggest that Menin may maintain exocrine pancreas homeostasis in response to inflammation and oncogenic stress [49]. To date, the putative biological function of Menin in the liver is not well defined. Menin's expression was found to be reduced in old mice, particularly in the white adipose tissue and liver of those with diabetes [8]. Menin regulates lipid deposition by interacting with the well-known transcription factor FoxO1 [41, 48, 50], which controls both glucose and lipid metabolism. Our previous results indicated that, in mouse hepatocytes, Menin binds to FoxO1, targeting the transcription of the glucose uptake rate-limiting enzyme GK and/or the fatty acid receptor CD36 [48]. Interestingly, FoxO1 expression is controlled by Menin in hepatocytes, with increased transcription upon Menin knockdown and decreased transcription upon Menin overexpression [48]. Therefore, Menin is believed to play an essential role in orchestrating metabolic homeostasis in hepatocytes, dynamically switching the metabolic pathway between glucose uptake and fatty acid uptake so as to maintain stable metabolic energy. This study aims to investigate the upstream mediator role of Menin in the liver and explore its function in maintaining metabolic homeostasis by examining its potential link to fatty liver disease. The results of the study revealed that Menin not only binds to FoxO1 but also recruits SIRT1 and PPARγ, enabling their translocation between the cytoplasm and nucleus. By doing so, Menin orchestrates glucose and lipid metabolism to maintain cellular energy balance in the liver.

## Results

### Menin regulates the expression of downstream target genes in PPAR signaling pathway by binding to PPARγ

**Fig. 1:**
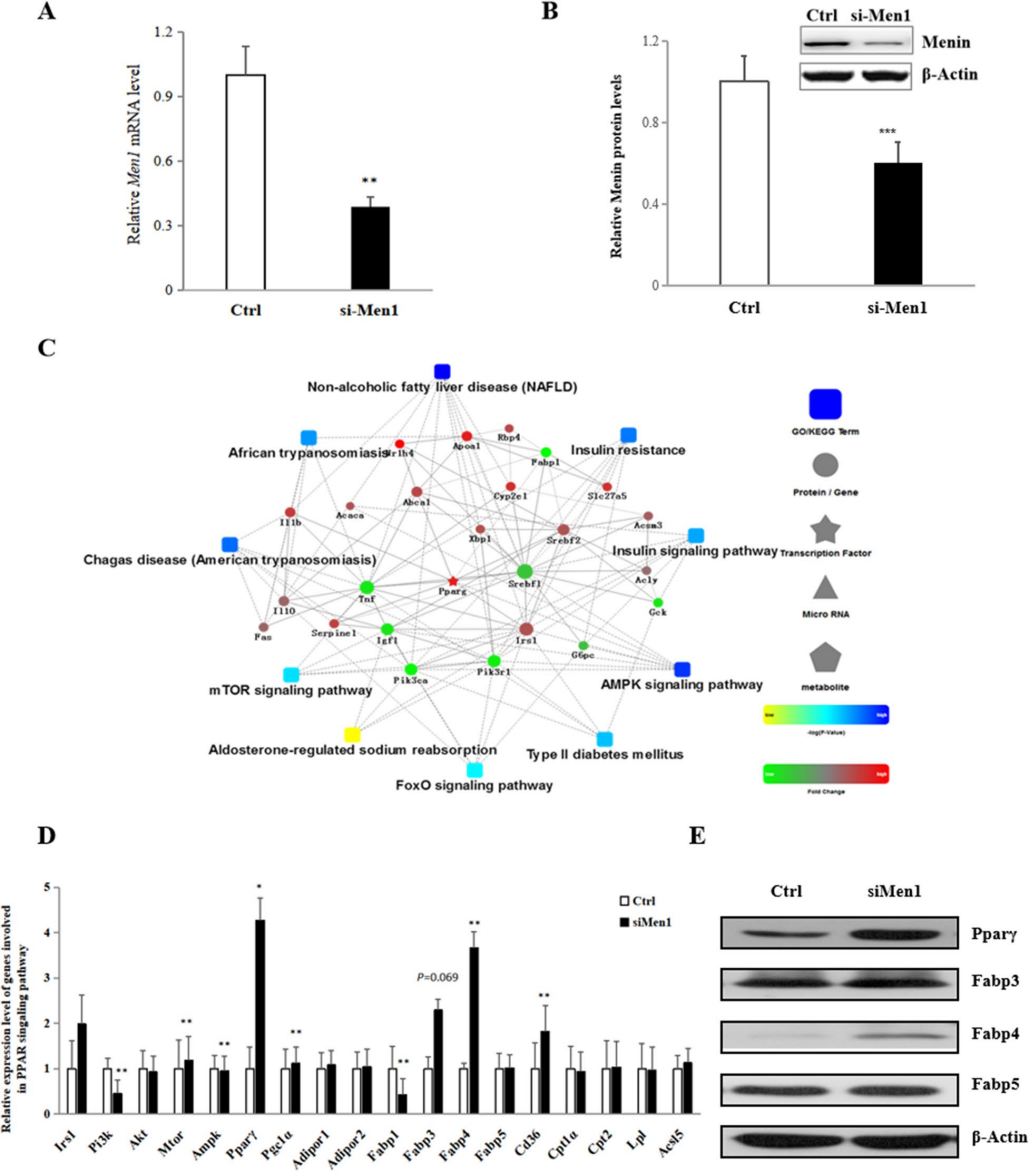
Menin knockdown in hepatocytes facilitated the expression of fatty acid transport-associated genes via the PPAR signaling pathway. **A**. Knockdown of Men1 mRNA was detected in mouse hepatocytes (NCTC-1469) at 24 h after transfection with Men1-specific siRNA (si-Men1) or non-specific negative control siRNA (Ctrl). **B**. Menin protein was suppressed in hepatocytes, as shown in the representative Western blot images presented in the inserted panel. **C**. Protein–protein interaction network analysis of differentially expressed genes detected using RT^2^. Profiler PCR Array in Menin lower-expressed hepatocytes indicates that lower Menin cause fatty liver disease or disorder in glycolipid metabolism via enriched signaling pathways such as NAFLD, AMPK signaling pathway, insulin signaling pathway, etc. **D**. Genes involved in the PI3K/Akt/mTOR, AMPK, and PPAR signaling pathways were regulated upon inhibition of Menin expression, which are downstream targets of Menin, such as enhanced *Fabp3*, *Fabp4*, and *Cd36*. **E**. Lower Menin expression in hepatocytes led to elevated protein expressions of PPARγ, FABP3, FABP4, and FABP5

Menin is highly expressed in metabolic tissues such as the liver, kidney, and pancreas (Additional file 2: Figure S1). Our previous study demonstrated that Menin has a critical role in regulating lipid deposition through the transcription factor FoxO1, which is essential for metabolic homeostasis regulation [48]. In this study, we investigated the hepatic molecular regulatory mechanism of Menin by examining gene expression in hepatocytes with lower Menin expression levels. A commercial RT^2^ Profiler PCR Array focused on fatty liver disease was used to analyze the gene expression. Of 76 genes from different pathways, 33 showed significant differential expression levels, and protein–protein interaction (PPI) analysis revealed enrichment in glucose metabolism (insulin signaling pathway, PI3K/Akt/mTOR signaling pathway, and FoxO signaling pathway) and lipid metabolism pathways (AMPK signaling pathway and receptors and transporters associated with PPAR signaling pathway), particularly in the non-alcoholic fatty liver disease (NAFLD) pathway (Fig. 1C). These findings were consistent with those from RNA-seq analysis upon Menin knockdown in hepatocytes [48]. We also confirmed that Menin regulates *Pi3k*, *mTor*, and *Ampk* expression levels (Fig. 1D), enhances downstream gene expressions in the PPARγ signaling pathway, such as *Fabp3*, *Fabp4*, and *Fabp5* (Fig. 1D, E), and inhibits the expression of the liver-specific FABP, *Fabp1* (Fig. 1D, Additional file 2: Figure S2 right panel), at both mRNA and protein levels. Moreover, Menin can regulate fatty acid uptake and lipid synthesis processes via the PPARγ signaling pathway [48]. Additionally, lower Menin expression induced inflammation, causing elevated expression of Il1α and Il1β (Additional file 2: Figure S3).

**Fig. 2:**
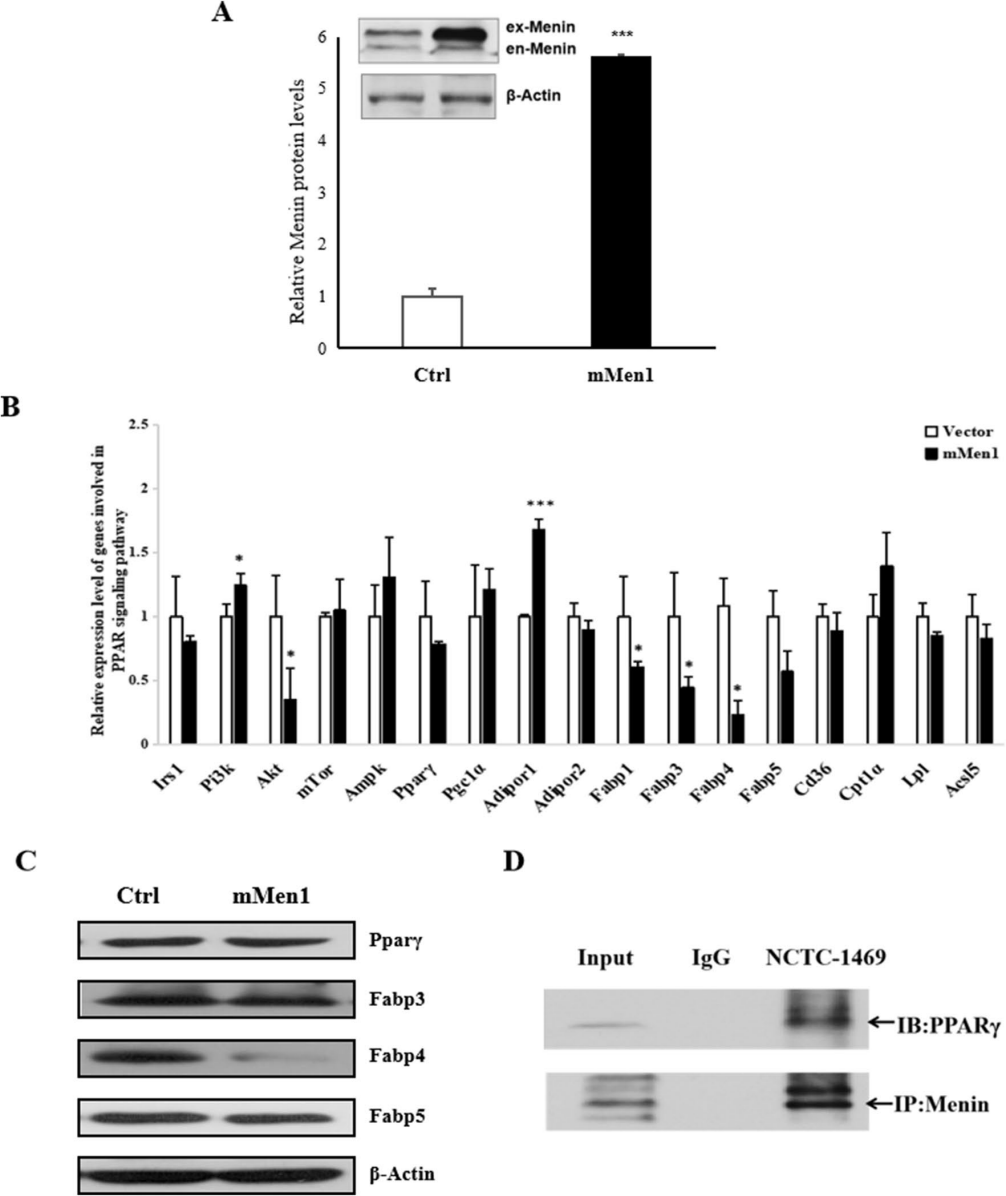
Higher Menin expression in hepatocytes suppressed fatty acid transport by interacting with PPARγ and targeting its downstream genes. **A**. Menin protein was over-expressed in hepatocytes at 24 h after transfection with pcDNA3.1-mMen1 (mMen1) or an empty vector (Ctrl). The representative Western blot images are shown in the inserted panel, where ex-Menin represents exogenously over-expressed Menin, and en-Menin represents endogenous Menin. **B**. Downstream target genes such as *Fabp3*, *Fabp4*, *Fabp5*, and *Cd36* were found to be inhibited upon Menin over-expression. *P < 0.05, **P < 0.01, *** *P* < 0.001. **C**. Higher Menin expression in hepatocytes inhibited the protein expression of PPARγ, FABP3, FABP4, and FABP5. **D**. Menin was found to interact with PPARγ in plain mouse hepatocytes via a Menin-IP assay

Conversely, Menin's transcriptional repressor effects on the PPARγ signaling pathway were observed in experiments with higher Menin expression levels (Fig. 2). The expression of *Fabp3/Fabp4/Fabp5* was inhibited (Fig. 2B, C), as well as *Fabp1* at both mRNA and protein levels (Additional file 2: Figure S2 left panel). These findings suggest that Menin plays a compensatory regulatory role in hepatic fatty acid uptake through other FABPs, although it does not directly control the expression of FABP1. This is consistent with our previous finding that higher Menin expression alleviates lipid accumulation in hepatocytes [48]. Moreover, we found that besides Menin's recruitment role of FoxO1, it also interacts with PPARγ in hepatocytes (Fig. 1E), indicating Menin's crucial and upstream regulatory role in hepatic lipid metabolism and its possible orchestration in maintaining metabolic homeostasis of hepatocytes by sensing cellular energy environment such as glucose and fatty acid concentration, and related hormones like insulin and PRL [33, 48].

### In an induced fatty hepatocyte model, along with elevated Menin expression, similar pathways were enriched, including PPARγ signaling pathway, NAFLD pathway, insulin signaling pathway

**Fig. 3:**
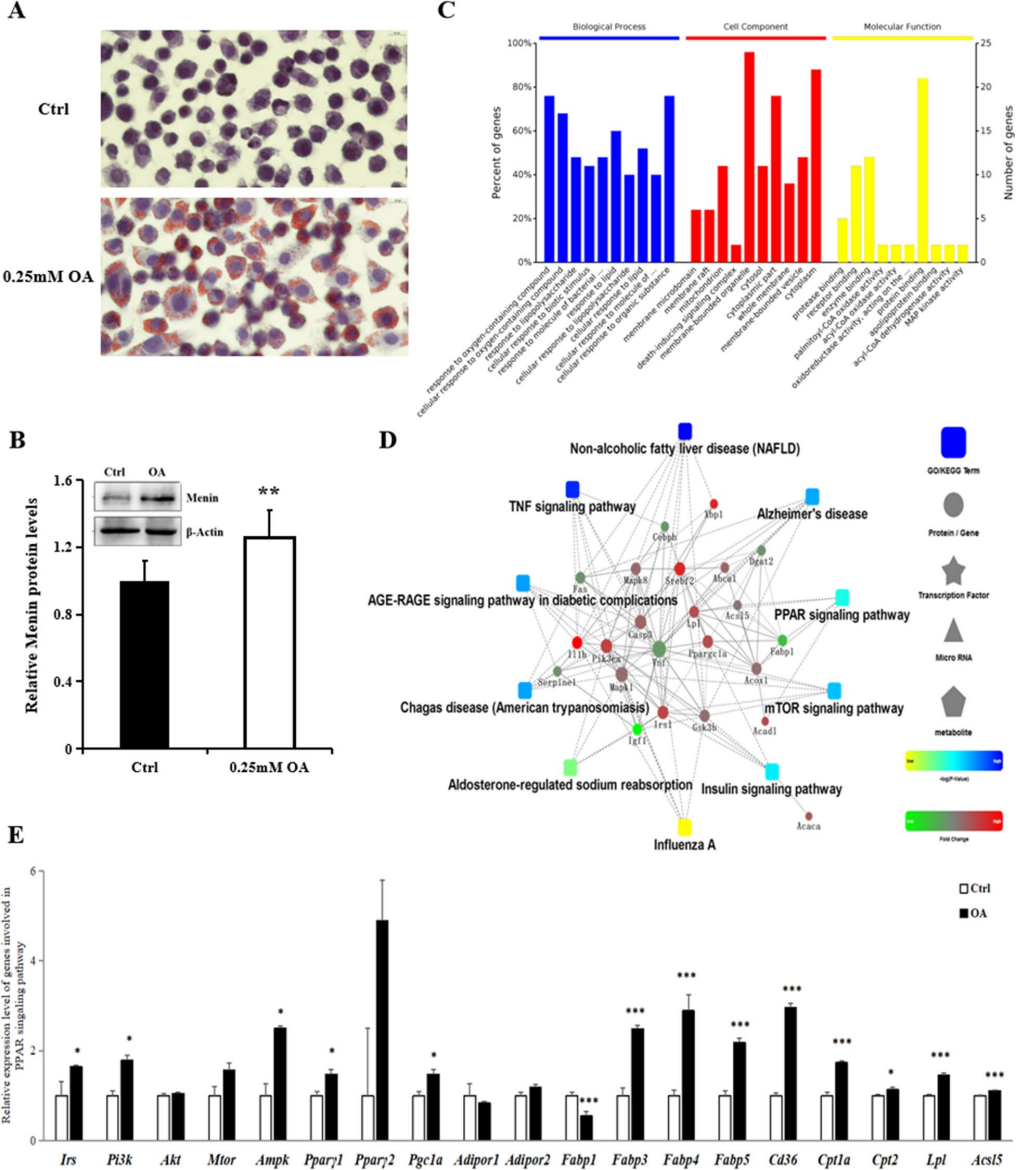
A fatty cell model was induced using sodium oleate (OA) and accompanied by enhanced fatty acid transport via the PPAR signaling pathway, similar to that of lower Menin expression. **A**. Sodium oleate (OA, 0.25 mM) was used to induce mouse hepatocytes into a fat-deposited cell model. Red oil staining indicated significantly increased TG (Additional file 2: Figure S2B), and lipid droplets accumulated in hepatocytes upon OA treatment. **B**. Both mRNA and protein levels of Menin were elevated in fatty hepatocytes upon OA induction. **C**. Gene ontology analysis of differentially expressed genes detected using RT^2^. Profiler PCR Array in OA-induced fatty hepatocytes. **D**. Protein–protein interaction network analysis of differentially expressed genes indicates fat deposition in hepatocytes upon OA treatment was accompanied by dysregulations in signaling pathways associated with fatty liver disease or disorder in glycolipid metabolism, such as NAFLD, PPAR signaling pathway, insulin signaling pathway, etc. **E**. The enhanced expression of a series of target genes suggested OA-induced fat accumulation in hepatocytes was regulated via the PPARγ signaling pathway, similar to that of Menin knockdown experiments, except for the elevated fatty acid transport genes *Fabp3/4/5*, which were supposed to be inhibited by elevated Menin. *P < 0.05, **P < 0.01, ***P < 0.001

To investigate the role of Menin in hepatocytes, we established a fatty cell model using 0.25 mM Oleic acid (OA) induction for 72 h. This model showed enhanced triglyceride synthesis (Additional file 2: Figure S4B) and lipid droplet appearance without loss of cell viability (Fig. 3A; Additional file 2: Figure S4A). Interestingly, Menin expression was found to be significantly increased upon OA induction (Fig. 3B). We analyzed the same set of genes associated with fatty liver and identified 36 genes with significant differences. Gene ontology analysis revealed that biological processes associated with lipid metabolism were enriched, such as response to lipopolysaccharide and response to lipid (Fig. 3C). PPI analysis of these differentially expressed genes revealed enrichment in the NAFLD pathway, as well as the PPAR signaling pathway, insulin signaling pathway, and mTOR signaling pathway, which all mediated the biological processes of lipid deposition (Fig. 3D). Importantly, similar genes targeted by Menin were observed in OA-induced hepatocytes, including enhanced expression of *Pi3k*, *mTor*, and *Ampk* and increased expression of genes promoting lipid synthesis through the PPARγ signaling pathway, such as *Pparγ*, *Fabp3/4/5*, *Cd36*, and *Cpt1*, except for inhibited *Fabp1* (Fig. 3E). The gene expression patterns upon OA induction in hepatocytes were similar to the regulatory pattern of Menin (Figs. 1 and 2), except for its suppressive regulatory role on genes associated with fatty acid uptake. Therefore, elevated Menin expression induced by OA (Fig. 3B) may play a role in attenuating fatty acid uptake (transport) to neutralize OA's promoting effect on lipid synthesis.

### Menin binds to the promoter regions of *Fabp3*, *Fabp4*, and *Fabp5* in a dosage-dependent manner

**Fig. 4:**
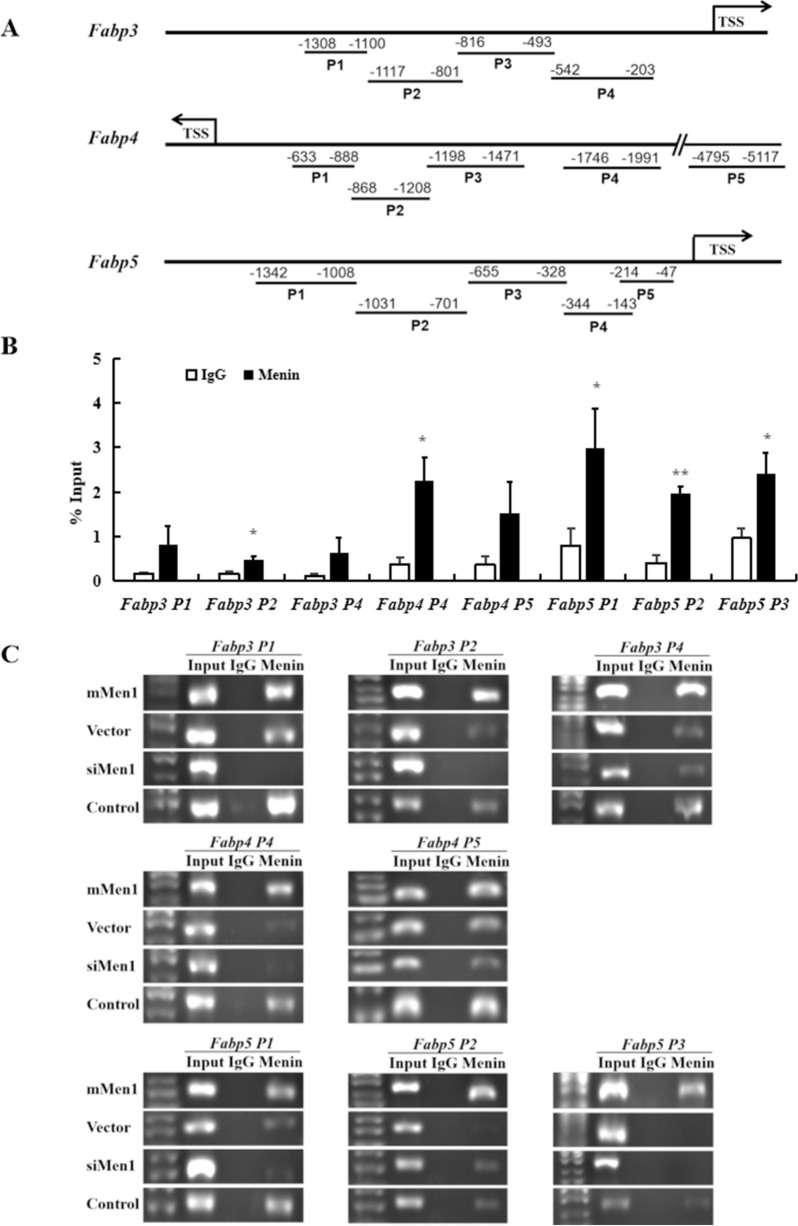
Menin binds to the promoters of fatty acid binding protein genes as the transcriptional targets of Menin in mouse hepatocytes. **A**. Several amplicons (P1, P2, etc.) were designed to detect the indicated promoter regions of the *Fabp3*, *Fabp4*, and/or *Fabp5* genes for Menin-ChIP assays. TSS represents the transcription start site, and curved arrows represent their transcription directions. **B**. Results of quantitative Menin-ChIP PCR assays in plain mouse hepatocytes from three independent Menin-ChIP experiments, showing the percentage of input chromatin bound by Menin, indicated that Menin occupied the promoter regions of downstream target genes of the PPAR signaling pathway, *Fabp3*, *Fabp4*, and/or *Fabp5*. *P < 0.05, **P < 0.01. **C**. Representative ChIP-PCR results of promoter regions of *Fabp3* (upper panel), *Fabp4* (middle panel), and *Fabp5* (lower panel) from anti-Menin ChIPs in Menin over-expression (mMen1) and Menin-knockdown (siMen1) hepatocytes, as well as their corresponding controls (Vector and Control)

To confirm Menin's regulatory role on fatty acid uptake genes Fabps, we conducted a Menin chromatin immunoprecipitation (ChIP) assay in plain hepatocytes and in hepatocytes with higher or lower Menin expression levels. We found that Menin bound to different promoter regions (Fig. 4A) of *Fabp3*, *Fabp4*, and *Fabp5* (Fig. 4B). Additionally, the amount of Menin binding to these promoter regions was dose-dependent and positively correlated with Menin expression levels (Fig. 4C).

### Further, Menin also cooperates with SIRT1 to imapct the expression of *Fabps*

**Fig. 5:**
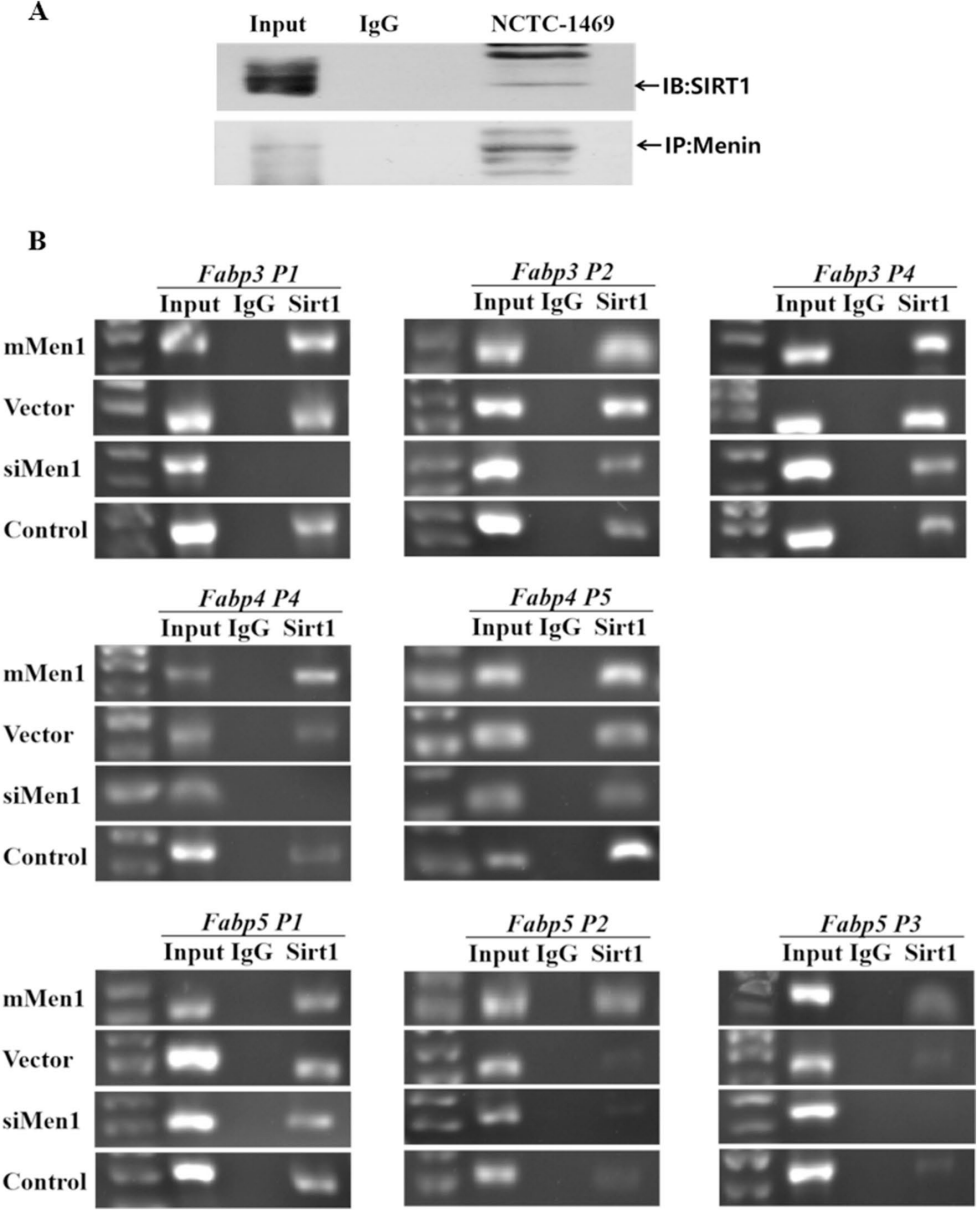
Histone deacetylase SIRT1 co-binds to the same promoter regions of *Fabp* genes being recruited by Menin. **A**. SIRT1 was found to interact with Menin in plain mouse hepatocytes via a Menin-IP assay. **B**. Representative ChIP-PCR results of the same promoter regions of *Fabp3* (upper panel), *Fabp4* (middle panel), and *Fabp5* (lower panel) from anti-SIRT1 ChIPs in Menin over-expression (mMen1) and/or Menin-knockdown (siMen1) hepatocytes, as well as their corresponding control cells (Vector and Control)

SIRT1 is a key deacetylase that plays a crucial role in regulating gene expression associated with energy metabolism by sensing the cellular concentration of NAD^+^/NADH and regulating mitochondrial biogenesis. In this study, we found that Menin also interacts with SIRT1 (Fig. 5A) in hepatocytes and co-binds to the same promoter regions of *Fabp3/4/5* (Fig. 5B). This finding further supports the notion that Menin plays an upstream regulatory role in orchestrating metabolic homeostasis by recruiting key transcription factors such as SIRT1, PPARγ, and/or FoxO1 and targeting fatty acid uptake and/or glucose uptake [48].

### The acting mode of Menin playing an orchestration role in metabolic homeostasis is also seen in fatty liver tissue of cattle, similar with that observed in fatty cell model

**Fig. 6:**
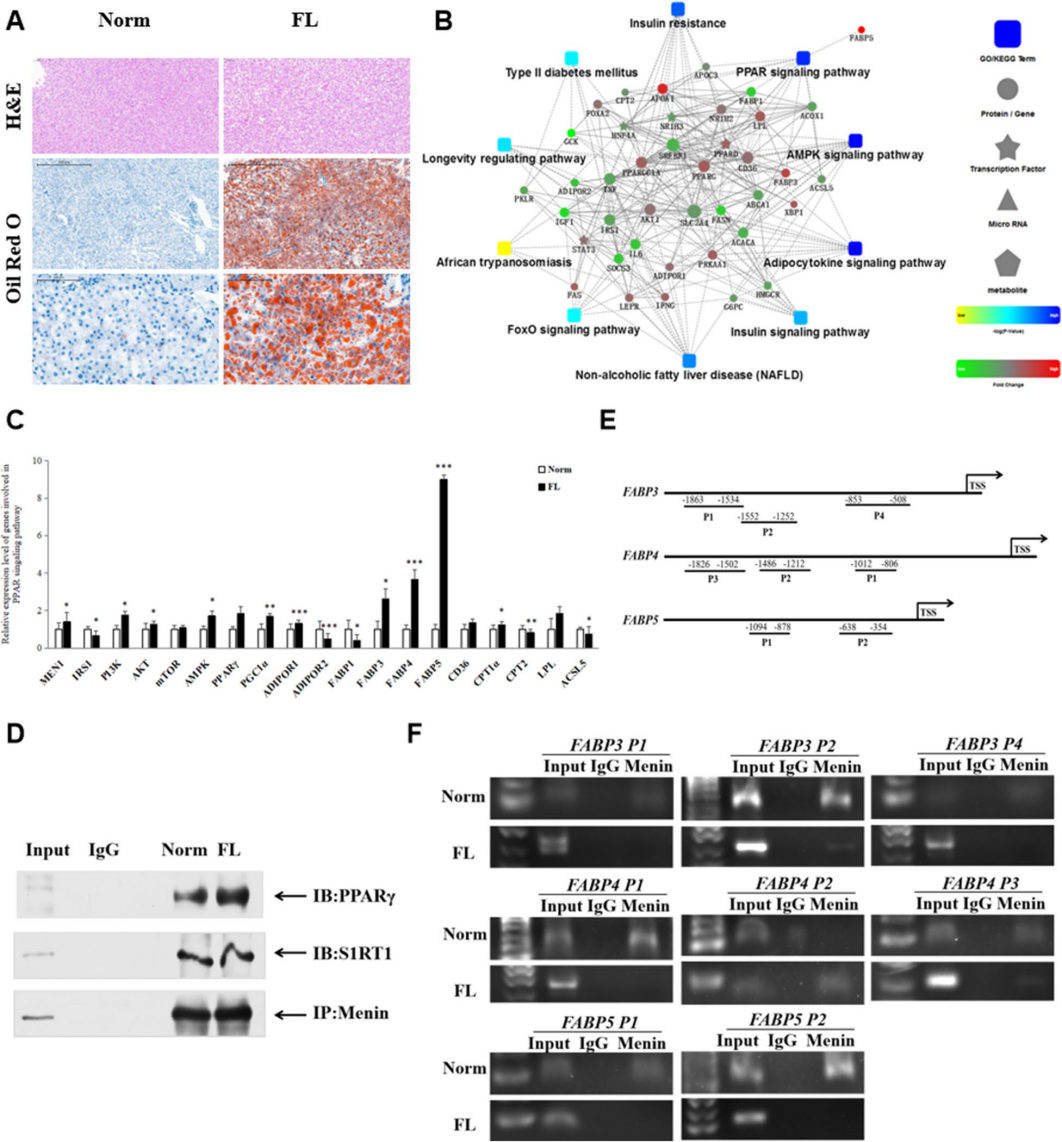
Elevated Menin is also observed in biopsied fatty liver tissues of dairy cows, and Menin occupies promoter regions of *FABPs*, acting as a transcriptional regulatory factor in hepatic lipid metabolism via the PPARγ signaling pathway. **A**. Representative hepatic histology sections of biopsied liver tissues from prenatal dairy cows. An Oil Red O staining assa, as well as Hematoxylin–Eosin staining (top panel), was used for fat content assessment in cells, thereby being classified into normal liver (Norm) and fatty liver (FL). Blue dots indicate cell nucleus, while brown or red indicates lipid droplets in cells that dissolved Oil Red. The bottom panel is a high-powered magnification of the central area of the middle panel. **B**. Protein–protein interaction network analysis of the differentially expressed genes associated with fatty liver disease, indicating enrichment in the PPAR signaling pathway, as well as NAFLD, AMPK signaling pathway, insulin signaling pathway, etc. **C**. Upstream and downstream target genes of the PPAR signaling pathway were upregulated in fatty liver of dairy cows compared to healthy ones (Norm). **D**. Elevated Menin in liver tissue was found to interact with PPARγ and SIRT1 via a Menin-IP assay. **E**. Several amplicons (P1, P2, etc.) were designed to detect the indicated promoter regions of the *FABP3*, *FABP4*, and *FABP5* genes for Menin-ChIP assays in liver tissues. TSS represents the transcription start site. **F**. Representative ChIP-PCR results of promoter regions of *FABP3* (upper panel), *FABP4* (middle panel), and *FABP5* (lower panel) from anti-Menin ChIPs in fatty liver (FL) and/or normal liver (Norm) from dairy cattle

To validate the cellular role of Menin, we conducted a systematic analysis using liver tissues biopsied from normal and fatty liver cattle (Fig. 6A). Overall, the gene expression pattern and cellular mechanism observed in the liver tissue were similar to what we observed in the fatty cell model upon OA induction (Fig. 3). The gene expression regulatory pattern was enriched in the non-alcoholic fatty liver disease pathway, insulin signaling pathway, FoxO signaling pathway, and PPAR signaling pathway, accompanied by lipid deposition in the liver (Fig. 6B, C). Notably, factors involved in the PPARγ signaling pathway showed enhanced expression in the fatty liver tissue, along with elevated Menin expression at both mRNA and protein levels (Fig. 6C; Additional file 2: Figure S5). Co-immunoprecipitation assays indicated that Menin recruited both SIRT1 and PPARγ in both normal and fatty liver tissues (Fig. 6D). In addition, Menin-ChIP assays validated that Menin bound to the promoter regions of *FABP3*, *FABP4*, and *FABP5* in liver tissues (Fig. 6E, F).

### Higher Menin in hepatocytes facilitated glucose uptake, but restained the fatty acid uptake and adipogenesis processes

To further confirm the mediator role of Menin in energy metabolism, we conducted a series of immunoblotting assays on hepatocytes with higher Menin expression. We found that higher Menin promoted the kinase activity of IRS1 (pIRS1(S307)/IRS1), GSK3β (pGSK3β(T216)/GSK3β), and AMPK (pAMPK(T172)/AMPK), while suppressing the activity of PPARγ (pPPARγ/PPARγ) and Akt (pAkt(S473)/Akt) (Fig. 6A, B). Consistent with these results on signaling pathways, higher Menin significantly enhanced the expression of glucose uptake protein GK and gluconeogenesis protein PCK (Fig. 6C, D), while inhibited the expression of fatty acid uptake protein CD36 (Fig. 6C) as well as FABP3/4/5 (Fig. 2). Therefore, Menin orchestrates glucose and lipid metabolism in hepatocytes to maintain metabolic homeostasis. However, in the OA-induced fatty cell model, the activity of these upstream factors regulated by Menin went in the opposite regulatory directions along with downstream factors such as CD36, GK, and PCK (Additional file 2: Figure S6), suggesting that OA treatment exceeds Menin's capacity to maintain cellular metabolic homeostasis, ultimately leading to excessive hepatic lipid deposition.

### Menin could suppressed the activity of Akt and PPARγ, but also deployed their cellular localization in nucleus and cytoplasm, along with the shuttling of SIRT1

**Fig. 7:**
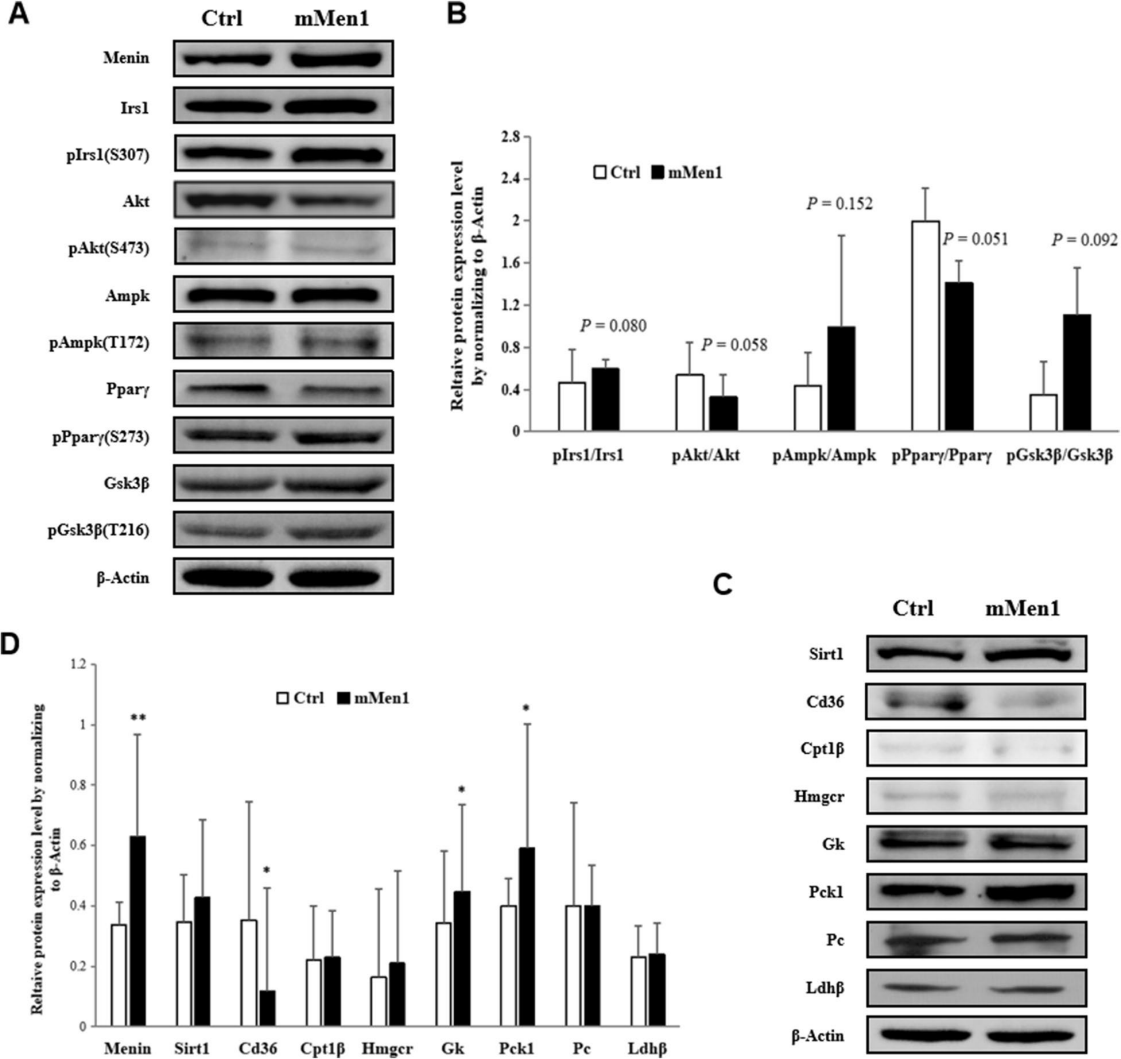
Higher Menin expression activates the insulin pathway and AMPK signaling pathway but inhibits the activity of Akt and PPARγ, facilitating glucose uptake and gluconeogenesis while suppressing fatty acid uptake and adipogenesis. **A**. Representative Western blot results of key mediator factors involved in the insulin receptor (activated IRS1 being phosphorylated at S307, and its total protein), Akt (activated Akt being phosphorylated at S437, and its total protein), AMPK (activated AMPK being phosphorylated at T172, and its total protein), PPARγ (activated PPARγ being phosphorylated at S273, and its total protein), and GSK3β (activated GSK3β being phosphorylated at T216, and its total protein) upon Menin overexpression in hepatocytes. **B**. Quantitative results of Western blot results obtained from three independent experiments, indicating enhanced activity of IRS1, AMPK, and/or GSK3β, but inhibited activity of Akt and/or PPARγ. **C**. Representative Western blot results of downstream factors involved in the PPAR signaling pathway upon Menin overexpression in hepatocytes, showing significantly inhibited fatty acid transporter CD36 but significantly elevated rate-limiting enzyme of glucose uptake GK and gluconeogenesis PCK. **D**. Quantitative results of Western blot results from three independent experiments, indicating that higher Menin expression suppresses fatty acid uptake (CD36) while facilitating glucose uptake (GK) and gluconeogenesis (PCK)

**Fig. 8:**
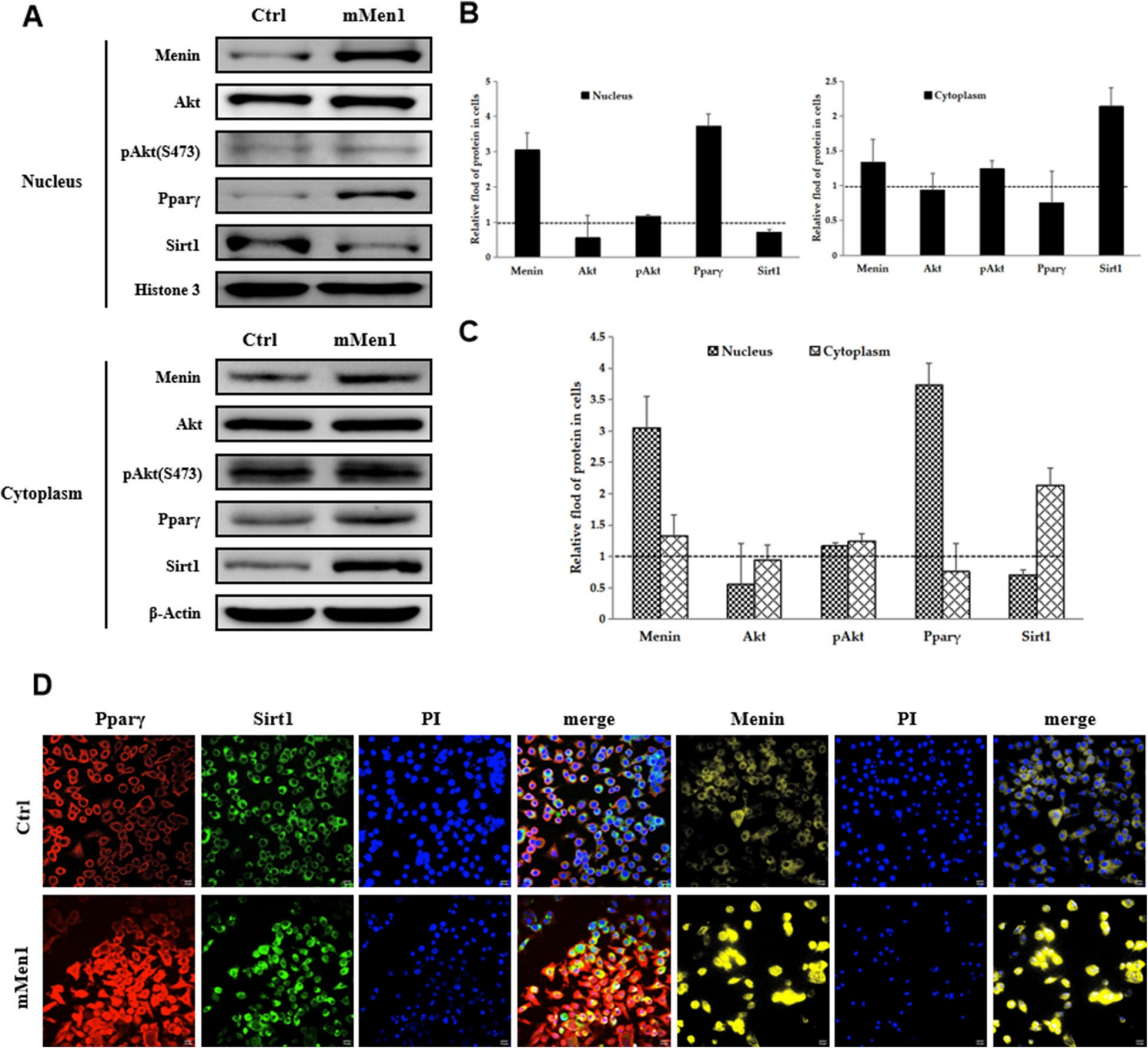
Menin restrains more activated Akt and translocates more SIRT1, but less PPARγ into the cytoplasm. **A**. The distribution proportion of Akt, PPARγ, and SIRT1 in the nucleus and cytoplasm are assessed upon higher Menin expression. Histone 3 and β-Actin are used as loading controls for the nucleus and cytoplasm extracts, respectively. **B**. Quantitative results of proteins in the nucleus (left panel) and cytoplasm (right panel). The dotted line at 1 represents equal proportional distribution, normalized by its negative control (Ctrl). **C**. Comparative results of concurrent distribution ratio of Menin, pAkt/Akt, PPARγ, and SIRT1 in the nucleus and cytoplasm of hepatocytes, suggest that Menin has similar distribution characteristics with activated Akt and PPARγ while opposite to that of SIRT1. **D**. immunofluorescence staining indicates that, compared to negative control (Ctrl), overexpressed Menin (yellow) promotes more PPARγ (red) translocate into the nucleus but expels more SIRT1 (green) into the cytoplasm

**Fig. 9:**
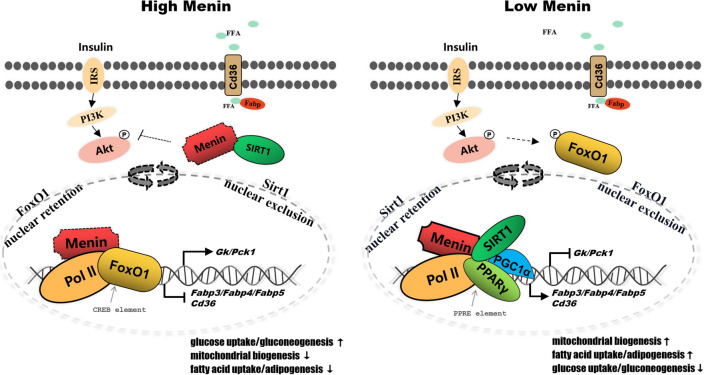
Schematic model of Menin recruiting transcriptional factors into promoters of *Fabps*, *Cd36*, and *Gk*, *Pck*, facilitating glucose uptake, gluconeogenesis, and mitochondrial biogenesis, while suppressing fatty acid uptake and adipogenesis through deploying the distribution ratio of activated Akt, PPARγ, and SIRT1 in the nucleus and cytoplasm. Dashed arrows indicate Menin carries its cargo and shuttles between cytoplasm and nucleus. *IRS* insulin receptor substrates, *FFA* free fatty acid, *FABP* fatty acid binding protein, *CD36* fatty acid receptor, *FoxO1* fork-head box O1, *SIRT1* sirtuin 1, *Pol II* RNA polymerase II, *GK* also named Gck, glucokinase, *PCK* also named PEPCK, phosphoenol-pyruvate carboxykinase, *PPARγ* peroxisome proliferator-activated receptor gamma, *PGC1α* PPARγ coactivator 1α

Menin has been found to respond to insulin concentration in hepatocytes, mediating hepatic lipid deposition [52],Wueacher et al., 2012; [48]. In addition to its role in mediating energy utilization, higher Menin was found to not only suppress the activity of Akt (S473) (Fig. 7A, B) but also retain activated Akt in the cytoplasm (Fig. 8A, B and C), thereby reducing its phosphorylation activity on FoxO1 [52] and facilitating the transcriptional regulatory role of FoxO1 in the nucleus on glucose metabolism-associated genes such as *Gk* and *Pck* (Fig. 7C, D, [48]. Conversely, Menin facilitated more PPARγ translocation into the nucleus by recruiting it and shuttling between the cytoplasm and nucleus (Fig. 8C, D). Interestingly, Menin concurrently enhanced the aggregation of SIRT1 in the cytoplasm (Fig. 8B, C and D), reducing its deacetylation role in the nucleus on the promoter regions of downstream target genes such as fatty acid transport genes *Fabp3/4/5*. As a result, higher Menin targeted the expressions of different downstream genes *Fabp3/4/5* and *Cd36* [48], suppressing fatty acid uptake and/or adipogenesis, while facilitating glucose uptake and/or gluconeogenesis (Fig. 9). Consequently, Menin played an orchestrating role in maintaining metabolic homeostasis by recruiting SIRT1, PPARγ, and/or FoxO1 to maintain cellular energy uptake and utilization (Fig. 9).

## Discussion

Fatty liver disease is a complex physiological process that involves lipid metabolism, insulin resistance, inflammatory response, and gluconeogenesis [29, 38, 45]. Investigating the relationship between hepatic metabolic homeostasis and fatty liver disease, a type of metabolic disorder, will refine our understanding of mechanisms in liver lipid and glucose metabolism and guide our investigation strategy in developing novel drugs for "metabolic dysfunction-associated fatty liver disease" (MAFLD) [15]. Liver specific ablation of *Men1* gene expression in healthy mice induces hepatic steatosis under high-fat dietary conditions [11], with significantly increased liver triglycerides coupled with increased liver steatosis and inflammatory markers, as well as increased serum insulin, glucose level and type 2 diabetic phenotype [53], suggesting hepatic menin is required for regulation of diet-induced glucose and lipid metabolism. Interestingly, the expression of Menin is reduced in the liver of aging mice. Hepatocyte-specific deletion of *Men1* induces liver steatosis in aging mice. Menin deficiency promotes high-fat diet-induced liver steatosis in mice [8]. In addition, reports from our lab and others demonstrate that Menin in hepatocytes can respond to surrounding insulin level and regulate the glucose uptake, but also mediate the lipid metabolism [48], with enhanced the TG accumulation and lipid deposition upon Menin knockdown in hepatocytes but alleviated upon Menin overexpression. Amazingly, Menin overexpression also alleviates the lipid deposition of OA-induced fatty hepatocytes. Mechanistically, Menin plays an essential mediator role in regulating hepatic metabolism via interacting with FoxO1 [48], the key transcription factor regulating both glucose and lipid metabolism. High levels of Menin suppress lipid accumulation, but low levels of Menin promote lipid accumulation in hepatocytes [48]. Here, we show that Menin also interacts with SIRT1, a critical regulatory node in metabolic processes, and PPARγ, a central factor targeting lipid metabolism and glucose homeostasis. Menin targets genes encoding rate-limited enzymes in glucose uptake (GK) and gluconeogenesis (PCK), as well as genes encoding fatty acid transporter proteins CD36 and FABPs. The dual functions of Menin in both glucose and lipid metabolism suggest its orchestration role in regulating hepatic homeostasis.

### Menin regulates hepatic glucose and fatty acid uptake via recruiting FoxO1/PPARγ/SIRT1 and deploying their translocation

Menin's ability to recruit factors (FoxO1/PPARγ/SIRT1) and shuttle between the cytoplasm and nucleus enables it to regulate cellular metabolic homeostasis. Our previous results indicated that glucose-mediated Menin inhibition occurs through the PI3K/Akt/FoxO1 signaling pathway [33]. In hepatocytes, Menin downregulates the level of active Akt and its kinase activity. By reducing the translocation of Akt from the cytoplasm to the plasma membrane (Fig. 8), Menin inhibits Akt-induced proliferation and antiapoptosis in non-endocrine and endocrine cells [47]. FoxO1 is a well-known key transcription factor that regulates cellular processes involved in glucose metabolism, apoptosis, oxidative stress, DNA damage repair, and cancer development. When Akt phosphorylates FoxO1, it mobilizes FoxO1 from the nuclei to the cytoplasm, leading to its inactivation and attunates its transcriptional impacts on downstream targets [41, 57]. Thus, under the condition of Menin overexpression, the recruitment amount of FoxO1 by Menin is reduced [48], as well as inhibited Akt expression and activity. The suppressed Akt activity allows FoxO1 to dephosphorylate and translocate from the cytoplasm into the nucleus, promoting glucose uptake and/or gluconeogenesis by targeting the expression of rate-limiting enzyme GK [48] and PCK, and inhibiting fat deposition by targeting the expression of fatty acid receptor CD36 [48] and transporter FABPs (Fig. 9). Moreover, FoxO1 can bind to the promoter region of *Men1* gene, enhancing its transcription level [57], suggesting a negative feedback loop between Menin and FoxO1 that allows them to orchestrate cellular homeostasis via deploying glucose and lipid metabolism. This action mode of Menin can also be reflected in insulin secretion and pancreatic islets β cell proliferation [42]. Loss of heterozygosity of Men1 gene in mouse embryonic fibroblasts, as well as specific knockout of *Men1* gene in pancreatic tissue, leads to β-Catenin aggregates in the nucleus, suppressing the phosphorylation of β-Catenin and activating the Wnt/β-Catenin signaling pathway [25]. When Menin expression level increases, the aggregation of β-Catenin in the nucleus decreases, resulting in decreased transcriptional activity and inhibiting pancreatic islets β cell proliferation [7].

Regarding the mechanism of the Menin-SIRT1/PPARγ pathway, our findings are consistent with previous reports [8, 48]. We found that Menin recruits histone deacetylase SIRT1 to regulate hepatic *Cd36* and *Fabp3/4/5* expression, mediating fatty acid uptake and TG accumulation by regulating the PPARγ pathway. SIRT1 is a NAD^+^-dependent deacetylase and cellular energy sensor involved in regulating the transcription of various genes and affecting lipid metabolism. Both Menin and SIRT1 are widely expressed in various tissues and cell types, and their interaction has diverse functions impacting energy metabolism, aging, or tumorigenesis [8, 18, 48]. Heterozygous deficiency of Sirt1 (Sirt1+/− mice) has been found to increase the expression of inflammation-related genes and reduce ApoB expression, leading to lipid accumulation in the liver and fatty liver [55]. Studies in mice have revealed that Menin interacts with the LLXXL domain on peroxisome proliferator-activated receptor (PPARα/γ) and regulates gene expression by binding to the peroxisome proliferators response element (PPRE) on the target gene, playing an essential role in adipocyte differentiation, fatty acid oxidation, de novo fat synthesis, and lipoma development [11, 14, 39]. Moreover, Menin can suppress the transcriptional activity of liver X nuclear receptor LXRα and physically interact with LXRα, thereby inhibiting the pathogenesis of fatty liver. Menin overexpression reduces the expression of LXRα target genes, including *Srebp-1c*, *Fasn*, and *Scd-1* [10]. PPAR pathways are linked to various diseases, including obesity, diabetes, cardiovascular disease, and cancer, because they belong to the nuclear hormone receptor superfamily, which are versatile receptors that contribute to adipogenesis, lipid and glucose metabolism, immune function, cell growth, and cell differentiation. Elevated Menin was found to suppress PPARγ activity (Fig. 7B), improving insulin resistance and attenuating the development of diet-induced NAFLD [4, 29]. FABPs, downstream targets of PPARγ, play a crucial role in lipid metabolism. Different types of FABP are expressed in various tissues with different abundance, and they play an important role in signal transduction pathways and are thought to be involved in fatty acid transport and lipid deposition. Higher levels of Menin significantly downregulate the expression levels of *Fabp3*, *Fabp4*, *Fabp5*, and *Cd36*, whereas lower levels of Menin enhance their expression levels, thereby mediating hepatic adipogenesis (Fig. 9).

The ability of FoxO1 and/or PPARγ in the nucleus to act as a transcription regulator depends on the expression level of Menin [48], which plays a role in its translocation. This is opposite to the distribution of SIRT1 in the cytoplasm and nucleus (Fig. 9). Acetylation of FoxO1 increases its phosphorylation levels at Ser253 through the PI3K/Akt signaling pathway, thereby reducing its affinity with target DNA [34]. Acetylation of FoxO1 is critical for regulating gluconeogenesis because it promotes FoxO1 nuclear exclusion and inhibits its binding to gluconeogenic promoters [32]. It is possible that there is a link in hepatocytes between Menin's orchestration role in maintaining metabolic homeostasis and SIRT1's function in regulating acetylation levels.

### FABP3, FABP4, and FABP5 are more readily deployed as compensatory factors for the deficiency of FABP1 upon hepatic metabolic disturbance or liver injury

It is noteworthy that in our study, FABP1 expression was found to be inhibited in all conditions, both in vitro and in vivo, with lower or higher levels of Menin (Figs. 1D and 2B), as well as in natural or induced fatty hepatocytes (Figs. 3E and 6C). FABPs are a group of intracellular chaperone molecules involved in lipid-mediated processes, which are considered key mediators in metabolic and inflammatory pathways. FABP1, also known as hepatic fatty acid binding protein or L-FABP, is a liver-specific FABP that plays important roles in intracellular lipid metabolism in liver and kidney. Studies have shown that FABP1 is closely related to the occurrence and development of diabetes [22]. It is also considered an early marker of diabetic nephropathy to predict the outcome of the disease due to its sensitive indicator of kidney injury [1, 2, 37]. Recently, H-FABP (FABP3) has emerged as another potentially promising biomarker for myocardial injury because it increases as early as 0–3 h from the onset of acute myocardial infarction [56, 58]. FABP4-deficient mice exhibit severe insulin resistance [21], and NAFLD is known to be associated with an increased risk of other extrahepatic complications, including type 2 diabetes, fatal and non-fatal cardiovascular diseases (CVD), as well as incident chronic kidney diseases (CKD) [29]. Our next point of interest is whether the expression level of FABP1 can be considered an early marker to predict the disturbance of metabolic hemostasis and/or hepatic injury [26], and what cellular mechanisms are causing suppressed FABP1 expression. Our preliminary results suggest that the suppression of FABP1 in hepatocytes attenuated TG accumulation and lipid deposition, likely rescuing the metabolic system balance (unpublished data).

Fatty acid binding proteins (FABPs) are crucial fatty acid carrier proteins that play an important role in fatty acid uptake and lipid metabolism. Based on our observations, when insulin resistance or fatty liver disease occurs, the expression of liver-abundant FABP1 is inhibited as a compensatory mechanism. Other types of FABPs, such as FABP3, FABP4, and FABP5, are more readily expressed upon liver injury to compensate for the loss of FABP1. For example, loss of FABP4 in adipocytes is compensated by over-expression of FABP5 [17]. In the study, resulting from higher levels of serum insulin [33, 48], the inhibited Menin switchs to targeting *Fabp3/4/5* gene expression leading to elevated fatty acid uptake, but attenuates its transcriptional activator role on glucose uptake genes *Gk*, *Pck* leading to inhibited glucose uptake.

### The multifaceted molecular functions of Menin make it a more suitable coordinator for cellular metabolic homeostasis

The elevated expression of Menin in fatty liver tissue and/or in an OA-induced fatty cell model indicates that Menin activation readily exhibits its compensatory function for hepatocyte and/or liver injury, striving to maintain cellular metabolic homeostasis. However, even though Menin’s expression was elevated upon the sustained fatty acid (OA) bursts in hepatocytes, Menin’s orchestration function is not strong enough to counteract the explosive damage from fatty acid transport and fat deposition, leading to metabolic disorders. This phenomenon is perfectly consistent with that observed in human, mouse, and/or cattle hepatic diseases [8, 48, 54], the highly expressed Menin in normal livers gradually reduces during aging, and the reduced Menin contributes to the development of hepatic steatosis in aged mouse livers. However, considering Menin as a tumor suppressor, the elevated Menin might also mediate hepatic MAFLD towards carcinogenesis progression, such as NASH (nonalcoholic steatohepatitis) and HCC (hepatocellular carcinoma), due to Menin's function in mediating inflammation (Additional file 2: Figure S3; [9, 20].

Other evidence also suggests Menin’s role in orchestrating glucose and lipid metabolism to maintain cellular energy balance. First, homozygous loss of exons 3–8 of the Men1 gene (Men1^*−/−*^) causes embryonic lethality in mice, while mice with heterozygous loss of Men1 (Men1^+/−^) develop features similar to those of the human disorder MEN1 syndrome, including increased insulin levels and decreased blood glucose levels [5, 6, 27]. Second, Menin is abundantly expressed in multiple tissues and organs (Additional file 2: Figure S1), sensitively responding to surrounding enviroment and playing a crucial mediator role in maintaining metabolic homeostasis [33, 48, 57]. Menin is a sensitive regulator of glucose metabolism that can respond to changes in glucose and/or insulin concentrations via the Akt pathway in various cell types. Apart from its role in insulin response, Menin can also respond to steroid hormones acting through nucleus receptors such as PRL and ER [33]. Furthermore, Menin can control insulin secretion in pancreatic β cells in a feedback loop, thereby regulating blood glucose levels [3, 43]. The absence of Menin can promote pituitary (anterior lobe) cell proliferation and induce increased secretion of PRL [6]. Moreover, Menin targets the expression of PRL and ER mRNA by binding to their promoter [24, 36] and catalyzes the activity of ER-α, affecting the development of the mammary gland [13, 24]. Third, Menin's molecular properties endow it with powerful and indispensable characteristics in orchestrating metabolic homeostasis. As a ubiquitous scaffold protein, Menin interacts with a variety of transcription factors regulating their downstream gene expression associated with cellular metabolic homeostasis, especially factors closely related to metabolic homeostasis such as Akt [47], SIRT1 [8], FoxO1 [48, 51, 52], and PPARs [14]. Additionally, Menin can shuttle between the nucleus and cytoplasm carrying its "goods," thereby deploying cellular metabolic homeostasis and maintaining energy balance at the body level. The mechanism that Menin activating as a intermediator connecting hepatic glucose uptake and fatty acid uptake and maintaining metabolism homeostasis benefits the extensive drug development focused on the Menin.

Recent extensive drug development focused on the Menin-MLL interaction [30, 44, 49] also suggests Menin's broad orchestration roles in deploying metabolic pathways, especially the Menin’s deploying model on FoxO1/PPARγ/SIRT1 would contribute to the drug discovery on the MAFLD. This coincides with the latest proposal that the American Diabetes Association guidelines in 2022 have proposed PPARγ agonists (pioglitazone) and glucagon-like peptide-1 (GLP1) receptor agonists for treating diabetes, NAFLD, or NASH [16, 29], in response to global health threats, possibily because Menin/SIRT1 mediated PPARγ acetylation plays a crucial role in orchestrating adipose plasticity and metabolic rhythms [19].

In summary, we have demonstrated a new action mode in which Menin orchestrates glucose metabolism and lipid metabolism in hepatocytes and/or liver tissues, thereby maintaining cellular metabolic homeostasis. By responding to changes in energy levels or hormone concentrations, Menin can switch its regulatory role by targeting the expression of rate-limiting enzyme genes controlling energy utilization (glucose or fatty acid uptake) via shuttling FoxO1, PPARγ, and/or SIRT1 between the cytoplasm and nucleus. In other words, Menin acts as a hinge connecting glucose uptake and fatty acid uptake, the two determinants of metabolic health. These findings suggest that Menin could be targeted for the treatment of hepatic steatosis, NAFLD or MAFLD, and even other hepatic diseases.

## Materials and methods

### Cell culture and cell transfer

Immortalized mouse normal (healthy) hepatocytes (NCTC-1469) were grown in DMEM/F12 containing 10% fetal bovine serum, penicillin–streptomycin liquid at 37℃ in an atmosphere of 5% CO_2_. Lipofectamine 2000 (Invitrogen) was used for transient transfections as previously described [48]. The recombinant expression plasmid of *Men1* (mMen1) was constructed into backbone vector pcDNA3.1( +) (Ctrl) according to the deposited *Mus musculus* Men1 sequence (NM_001168489). Men1-specific siRNA (si-Men1) was designated and synthesized by Ribobio (Guangzhou, China), as well as its negative control (Ctrl).

### Animal experiments

All animal experiments were carried out according to the Regulations for the Administration of Affairs Concerning Experimental Animals published by the Ministry of Science and Technology, China (2004), and were approved by the Shandong Agricultural University Animal Care and Use Committee (approval number, SDAUA-2017-044).

### Total RNA extraction and qPCR analysis

Total RNAs were extracted from liver tissues (about 20 ng) or NCTC1469 cells (5 × 10^5^ cells) using an RNA extraction kit, followed by treatment with DNase I. The quality of total RNA was assessed through agarose gel electrophoresis and the calculation of OD_260_/OD_280_. Oligo(dT)-primed first-strand cDNA was employed for quantitative real-time RT-PCR (qRT-PCR) using SYBR-Premix Ex Taq II and an Mx3000p cycler. Each CT value was obtained from the averaged CTs of triplicate reactions. The expression level of target gene was normalized to its corresponding internal control β-actin. The data were plotted as fold change over their corresponding controls in cells (Ctrl) or health liver tissues (Norm). The 2^−ΔΔCT^ method was used to calculate the relative expression abundance of target mRNA.

### RT2 Profiler PCR array

Total RNA extracted from higher Menin (mMen1) and/or lower Menin (si-Men1) transfected cells was then transcribed into complementary DNA using PrimeScript RT Reagent Kit with gDNA Eraser (TaKaRa Bio). A total of 76 genes related to fatty liver disease were examined by RT-qPCR with the RT2 profiler PCR array kit (cat No. PAMM-157Z, Qiagen). Primers were provided by the kit and embedded in the 96-well plate. While, as for the gene expression detection of fatty liver tissues in cattle, primers were redesigned and and synthesized by Sangon Biotech (listed in Additional file 1: Table S2). The results are representative of at least three independent experiments to determine the statistical signifcance.

### Preparation of cell lysates

The frozen liver tissue samples were crushed in liquid nitrogen and then homogenized and lysed in RIPA buffer. The lysates were then centrifuged at 12,000 rpm for 15 min at 4℃, and the supernatant was collected in a fresh tube. Cultured plain or transfected mouse hepatocytes after 24 h of transfection were harvested and lysed with ice-cold cell lysis buffer and the supernatant was collected following centrifugation. Cytoplasmic and nuclear extracts were extracted according to the Manufacturer's guidance.

### Electrophoresis and immunoblotting

The concentration of tissue extracts or whole cell extracts were quantified by the BCA kit. Equal amounts of protein (20 μg) from each sample were separated by 10% SDS-PAGE and transferred to the PVDF membrane using 200 mA of constant current. Primary antibodies against Menin, other (phosphorylated) proteins and/or β-actin were used at 1:1000 dilution. The detailed inforamtion about the antibodies for detected proteins was listed in “Key Resources Table” (Additional file 1: Table S1). Antibodies against β-actin or Histone 3 were used as loading control for whole-cell/cytoplasmic extracts or nuclear extracts, respectively. The HRP-conjugated secondary antibody was diluted by 1000 as the working solution. Chemiluminescence detection was performed using BeyoECL Plus. The data are shown as the expression level normalized to their corresponding negative controls. The results are representative of three independent experiments that were used to determine the statistical significance.

### Fatty hepatocyte induction

Sodium Oleate (OA) treatment was used to induce NCTC1469 cells into fatty hepatocytes according to our previously described method [48], with significantly increased triglyceride accumulated and lipid droplets. Treatment conditions of OA for fatty liver cell model induction were optimized via gradient concentrations of OA (0.1 mM, 0.15 mM, 0.2 mM, 0.25 mM, 0.3 mM, 0.4 mM and 0.5 mM) treatment and/or time course (24 h, 48 h and 72 h) experiments (Additional file 2: Figure S4A and B). Eventually, treatment of 0.25 mM OA for 72 h was the optimum condition. In brief, cells were inoculated to a 96-well plate (1 × 10^4^ cells/well) and treated with 0.25 mM OA for 72 h, then followed by quantitation of cellular triglyceride (TG) content and Oil Red O staining.

### Cellular triglyceride (TG) assay

The level of TG in the cells was detected by ELISA. The cells were collected and disrupted by ultrasonication. Part of the cell lysates were detected protein concentration by BCA (Beyotime), and the other cell lysates were used for determination of TG using the Triglyceride assay kit (Nanjing Jiancheng). TG content was calculated as previously described [48]. Samples comprised the transfected control group and the treatment group. The blank was 1 × PBS. The calibration product was provided as part of the kit.

### Oil Red O staining of fatty liver tissue

The suspected dairy cattle after parturition within 2 weeks examined by serum biochemical traits were liver biopsed as previouly described [59], so as to assess the percentage of fat deposited cells in liver and thereby diagnose with fatty liver disease (FL) or normal liver (Norm), with the percentage of cells contained lipid drops 82.75% ± 2.13% (n = 5) and 5.66 ± 3.13% (n = 5), respectively.

### Immunoprecipitation assays

Immunoprecipitation (IP) experiments in plain hepatocytes and liver tissues were performed using Protein G Agarose Immunoprecipitation Kit (KPL) according to the Manufacturer's guidance.

### Chromatin immunoprecipitation assay

Chromatin immunoprecipitation (ChIP) experiments in plain, transfected cells and/or liver tissues were performed using Magna ChIP G-Chromatin Immunoprecipitation Kit (Merck millipore) according to the Manufacturer's guidance. The primers used for ChIP-PCR from cell extracts and liver tissue extracts were listed in Additional file 1: Tables S3 and S4, respectively.

### Histology analysis

Liver tissue samples were immediately snapfrozen in liquid nitrogen for later analysis or fixed in 5% polyformaldehyde for Oil Red O staining and HE staining. Briefly, after dipping the slides in 60% isopropanol for 1 min, they were stained in working Oil-Red-O solution and/or Eosine solution for 15 min, followed by treatment with 60% isopropanol for 1 min. After dipping in distilled water, the slides were counterstained with hematoxylin for 1 min and then washed with distilled water again, followed by mounting with the aqueous medium. Digital images of sections were acquired using Olympus BX51 microscope with Pro rec C3 software. The biopsied dairy cattle had their fatty liver condition diagnosed according to the average percentage of hepatic cells containing lipid droplets in the tissue.

### Immunofluorescence staining

The Menin overexpressed hepatocytes were cultivated on a cell slide in a 24-well plate until the clone reached the proper density and size had grown to a suitable size. Then, the culture medium was aspirated, and the cells were washed with DPBS, fixed at room temperature for 2 h, permeabilized with 0.1% Triton X-100 solution for 15 min at room temperature, blocked with a 3% BSA solution for 1 h at room temperature, incubated with primary antibody and secondary antibody for 1 h, and incubated with DAPI nuclear-staining solution for 5–10 min. Then, anti-fluorescence quenching solution was dropped on each slide. Each cell slide was placed face down, onto a glass slide, and finally sealed with nail polish. The slides were scanned and imaged with a Leica TCS SPE confocal microscope.

The following primary antibodies were used: anti-Menin (Bethyl Laboratories, A300-105A), anti-Pparγ (Bioss, bsm-33436 M), anti-Sirt1 (CST, #2496 T). The following secondary antibodies were used: goat anti-mouse IgG H&L Cy5 (Bioss, 0296G-Cy5), goat anti-rabbit IgG H&L PE (Bioss, bs-0295G-PE) and goat anti-rabbit IgG H&L FITC (Bioss, bs-0295G-FITC).

### Quantification and statistical analysis

The results are expressed as mean ± SD of at least three independent experiments. The difference was statistically analyzed using SAS 8.2 statistical software (SAS Institute, Cary, NC) for single factor analysis of variance (ANOVA). Significant difference was denoted *P* < 0.05 (*) and *P* < 0.01 (**).

### Supplementary Information

**Additional file 1: Table S1.** Key resources used in the study. **Table S2.** The primer information designed for mRNA expressing detection in the liver tissues of dairy cows. **Table S3.** The primers information used for Menin-ChIP and/or SIRT1-ChIP assays in the mouse hepatocytes, designed in the promoter region of PPARγ targeted genes. **Table S4.** The primers information used for Menin-ChIP assays in the liver tissues of dairy cows, designed in the promoter region of PPARγ target genes.

**Additional file 2: Figure S1.** Menin was abundently expressed in metabolism-associated tissues in cattle. A. Real-time quantitative PCR results of *MEN1* gene in different tissues of calves. The same data with the same letter is not significantly different (*P* > 0.05), and the different letters are the same (*P* < 0.05). B. Western blot results of Menin protein in different tissues of calves. The same data with the same letter is not significant (*P *> 0.05), and the different letters with the top is significant (*P *< 0.05). C. Immunohistochemical staining of Menin in different tissues of calves. The letters A, B, C, D, E and F at lower right corners represents the immunity histochemical staining results from liver, kidney, pancreas, testis, duodenal mucosa and duodenal muscle layer, respectively. **Figure S2.** Liver-specific fatty acid transporter Fabp1 in hepatocytes was found inhibited upon Menin higher- and/or lower expression. The protein expression of Fabp1 was shown to be suppressed in both conditions of Menin over-expression by transfecting Men1 cds clone (mMen1) and Menin low-expression by transfecting Men1 specific siRNA (si-Men1), compared with their negative controls (Vector and control) transfected cells. **Figure S3.** Menin knockdown induced inflammation in hepatocytes. The expresssion of inflammation promoting factors Il1α and Il1β were detected to be enhanced in Menin-specific siRNA (si-Men1) treated mouse hepatocytes (NCTC-1469) at 24 h after transfection, compared with that of nonspecific negative control siRNA (Ctrl) treated cells. **Figure S4.** Optimization of treatment conditions for fatty liver cell model induction using sodium oleate (OA). A. The viability of NCTC-1469 cells was measured under different concentrations of OA, indicating that 0.25 mM OA was the optimum condition; B. Upon treatment of NCTC-1469 cells with 0.25 mM OA for 24 h, 48 h and 72 h, the accumulate TAG (triglyceride) in cells was significantly increased, with lipid droplets appearance. **Figure S5.** Menin expression was elevated in fatty liver tissues of dairy cows, compared with that in normal livers. A. WB results of Menin protein expression in biopsied normal (Norm) and fatty liver (FL) tissues from different prenatal dairy cows. B. Quantitative results of WB results indicated Menin expression was elevated in biopsied fatty liver tissues. **Figure S6.** OA induction, producing a fatty cell model, activates insuling pathway and Akt, but inhibites the activity of Ampk, Pparγ and Gsk3β, enhancing fatty acid uptake and adopogenesis, but suppressing glucose uptake and glugluconeogenesis. A. Representative WB results of key mediator factors insulin receptor (activated Irs1 being phosphorylated at S307, and its total protein), Akt (activated Akt being phosphorylated at S437, and its total protein), Ampk (activated Ampk being phosphorylated at T172, and its total protein), Pparγ (activated Pparγ being phosphorylated at S273, and its total protein) and Gsk3β (activated Gsk3β being phosphorylated at T216, and its total protein), upon OA induction in hepatocytes. B. Quantitative results of WB results obtained from OA treated whole cell extracts, indicating that enhanced activity of Irs1, Akt, but inhibited activity of Ampk and Pparγ and/or Gsk3β. C. Representative WB results of down-stream factors involved in Ppar signaling pathway upon OA treatment in hepatocytes, showing increased fatty acid receptor Cd36, but inhibited rate-limit enzyme of glucose uptake Gk and gluconeogenesis Pck, Gsk3β. D. Quantitative results of WB results from OA treated whole cell extracts, indicating that OA induction facilitates fatty acid uptake (Cd36) and lipid synthesis, whileas suppresses gucose uptake (Gk) and glugluconeogenesis (Pck).

## Data Availability

The data associated with this paper are available upon reasonable request to the corresponding author. Reagents, antibodies and resources are listed in the supplemental table.

## Abbreviations

AMPK: Adenosine 5’-monophosphate (AMP)-activated protein kinase
Akt: Protein kinase B
CD36: Fatty acid receptor
CPT1β: Carnitine palmitoyltransferase 1β
ER: Estrogen receptor
FoxO1: Forkhead box O1
GSK3β: Glycogen synthase kinase-3β
FABP1: Liver fatty acid binding protein, L-FABP
FABP3: Heart fatty acid binding protein, HFABP
FABP4: Adipocyte fatty acid binding protein, A-FABP
FABP5: Epidermal fatty acid binding protein, E-FABP
GK: Glucokinase
GLP1: Glucagon-like peptide-1
HCC: Hepatocellular carcinoma
HMGCR: 3-Hydroxy-3-methylglutaryl coenzyme A reductase
IL1α: Interleukin 1α
IL1β: Interleukin 1β
IRS1: Insulin receptor 1
MAFLD: Metabolic dysfunction-associated fatty liver disease
NAFLD: Non-alcoholic fatty liver disease
NASH: Nonalcoholic steatohepatitis
OA: Oleic acid
PCK: Phosphoenolpyruvate carboxykinase
PGC1α: Peroxisome proliferator activated receptor γ coactivator 1α
PPARα: Peroxisome proliferator activated receptor α
PPARγ: Peroxisome proliferator activated receptor γ
PPRE: Peroxisome proliferators response element
SIRT1: Surtuin 1
IP: Immunoprecipitation
ChIP: Chromatin immunoprecipitation
LDHβ: Lactate dehydrogenase
mTOR: Mammalian target of rapamycin
NAD: Nicotinamide adenine dinucleotide
NADH: Reduction state of nicotinamide adenine dinucleotide
PI3K: Phosphoinositide 3-kinase
PC: Pyruvate carboxylase
PRL: Prolactin
