# Supplementary material for: Menin orchestrates hepatic glucose and fatty acid uptake via deploying the cellular translocation of SIRT1 and PPARγ
Source: Cell Biosci. 2023 Sep 22;13:175. doi: 10.1186/s13578-023-01119-y (PMC10517496; doi:10.1186/s13578-023-01119-y)
Supplement: Supplementary file 1 — Additional file 1: Table S1. Key resources used in the study. Table S2. The primer information designed for mRNA expressing detection in the liver tissues of dairy cows. Table S3. The primers information used for Menin-ChIP and/or SIRT1-ChIP assays in the mouse hepatocytes, designed in the promoter region of PPARγ targeted genes. Table S4. The primers information used for Menin-ChIP assays in the liver tissues of dairy cows, designed in the promoter region of PPARγ target genes. [file 13578_2023_1119_MOESM1_ESM.docx]

**Table S1 Key resources used in the study**

| **Reagent or Resource** | **Source** | **Identifier** |
| --- | --- | --- |
| Antibodies | | |
| Menin antibody for WB | Bethyl | A300-105A |
| Menin antibody for ChIP | abcam | Ab31902 |
| Akt | Cell signaling Technology | 4691 |
| Phospho-Akt (S473) | Affinity Biosciences | AF0908 |
| Irs1 | Bioss Antibodies | bs-0172R |
| Phospho-Irs1 (S307) | Bioss Antibodies | bs-2736R |
| Pparγ | Bioss Antibodies | bs-3737R |
| Phospho-Pparγ (S273) | Bioss Antibodies | bs-4888R |
| Ampk | Cell signaling Technology | 5831T |
| Phospho-Ampk (T172) | Cell signaling Technology | 2535T |
| Gsk3β | Bioss Antibodies | bs-7332R |
| Phospho-Gsk3β (T216) | Bioss Antibodies | bs-4079R |
| Fabp 1 | Proteintech Group | 13626-1-AP |
| Fabp 3 | Proteintech Group | 10676-1-AP |
| Fabp 4 | Proteintech Group | 12802-1-AP |
| Fabp 5 | Proteintech Group | 12348-1-AP |
| Gk | Bioss Antibodies | bs-1796R |
| Pck | Bioss Antibodies | bs-4972R |
| Hmgcr | Bioss Antibodies | bs-5068R |
| Cd36 | Bioss Antibodies | bs-1100R |
| Cpt1β | Bioss Antibodies | bs-5045R |
| Pc | Proteintech Group | 16588-1-AP |
| Ldhβ | Proteintech Group | 14824-1-AP |
| SIRT1 for WB | Cell signaling Technology | 2496T |
| SIRT1 for ChIP | Merk | 04-1557 |
| PPARγ | Santa | sc-7273 |
| Histone 3 | RuiYingBio | RLM3038 |
| β-actin | Beyotime | GB12001 |
| Anti-Rabbit HRP | Servicebio | GB23303 |
| Anti-mouse HRP | Servicebio | GB23301 |
| Bacterial strains | | |
| *Escherichia coli* Bl21 (DE3) strain | TransGen Biotech | Cd601-02 |
| *Escherichia coli* Dh5α strain | TransGen Biotech | C502-03 |
| Chemicals, peptides and reagents |  |  |
| RIPA lysis buffer | Beyotime | P0013C |
| PMSF | sigma | P7626 |
| 1×DPBS basic | Gibco | C14190500BT |
| DMEM/F12 | Gibco | C11330500BT |
| Penicillin Streptomycin solution | Gibco | 15140-122 |
| Trypsin-EDTA (0.25%) | Gibco | 25200-072 |
| Opti-MEM I Reduced Serum Medium | Invitrogen | 31985062 |
| Lipofectamine 2000 reagent | Invitrogen | 11668027 |
| Fetal bovine serum | Gibco | 16000-044 |
| SDS-PAGE Transfer Buffer | Servicebio | G2017 |
| SDS-PAGE Running Buffer | Servicebio | G2018 |
| Tris Buffered Saline (TBS) | Servicebio | G0001 |
| Tween-20 | Solarbio | T8220 |
| T4 DNA ligase | NEB | M0202S |
| Sodium Oleate | Sigma | 07501-250MG |
| DMSO | Sigma | D2650-100ML |
| EDTA | Sigma | 3690 |
| Critical commercial assays | | |
| BeyoECL plus kit | Beyotime | P0018S |
| BCA kit | Beyotime | P0012S |
| Nuclear and Cytoplasmic Protein Extraction Kit | Beyotime | P0027 |
| RNA extraction kit | TIANGEN | DP430 |
| SYBR green-PCR kit | GenStar | A303-05 |
| EZ ChIP kit | Millipore | 17-371-RF |
| PurePlasmid Mini Kit | CWBIO | CW0500M |
| All-in-one RT MasterMix | abmGood | G492 |
| Oil Red O staining kit | Jiancheng Biotech | D027-1-1 |
| Triglyceride Assay kit | Jiancheng Biotech | A110-1-1 |
| RT2 Profiler PCR Array | QIAGEN | PAMM-157Z |
| Experimental models: Cell lines | | |
| NCTC1469 | China Intrastructure of Cell Line Resource | NCTC1469 |
| Experimental models: organisms | | |
| Perinatal cows | This study | Local farms |
| oligonucleotides | | |
| Men1 siRNA CCACTGTTATCCAAGACTA | RIBOBIO | siG12315132207 |
| Control siRNA | RIBOBIO | No public sequence ^*^ |
| Oligonucleotides for qTR-PCR | This study | Table S1 |
| Oligonucleotides for Menin-ChIP in cells | This study | Table S2 |
| Oligonucleotides for SIRT1-ChIP in cells | This study | Table S2 |
| Oligonucleotides for Menin-ChIP in tissue | This study | Table S3 |
| Recombinant DNA | | |
| pcDNA3.1(+)-mMen1 | This study | N/A |
| Software and algorithms | | |
| SAS 8.2 | SAS Institute Inc. |  |
| ImageJ version 1.8 | Schneider et al., 2012 | https://imagej.nih.gov/ij |
| Image- Pro Plus 6.0 | MEDIA CYBERNETICS |  |
| GraphPad Prism 8.0.2 (263) | GraphPad Software |  |

**Table S2. The primer information designed for mRNA expressing detection in the liver tissues of dairy cows.**

| **Gene** | **Primer sequence（5'-3'）** | **Product size** | **Gene** | **Primer sequence（5'-3'）** | **Product size** |
| --- | --- | --- | --- | --- | --- |
| *ABCA1* | F：GCGTGGCGTGGCAAGACTAC | 186 | *APOB* | F：TCAAGATTGACGGACAGTTCAGAGC | 179 |
|  | R：AGACGGAGGTGGTGAGGTTGAAG |  |  | R：GGAGACAGTGCCATCATCATACCTTC |  |
| *ABCG1* | F：CCAAGTCAGTGTGCGTCTCAGTG | 107 | *APOC3* | F：CTGCTCCTTCTTGCTGCCTTCC | 125 |
|  | R：ATTGTTGTCCTCCTTCTTCAGATGTCC |  |  | R：GCATCCTTGGCGGTCTTGGTG |  |
| *ACACA* | F：GCTATGGAAGTCGGCTGTGGAAG | 108 | *APOE* | F：CGACATGGAGGACTTGCGGAAC | 158 |
|  | R：TCGTCAGGAAGAGGCGGATGG |  |  | R：GGCGCTTCTTCAGGTCGTCAG |  |
| *ACADL* | F：TTGAAGATGTACGGTTGCCAGCAG | 82 | *ATP5C1* | F：TGATGCTGATGTGCTGCGGAAC | 87 |
|  | R：TGTGGAAGCTCTTGCATGAGGTAATAG |  |  | R：TGTCCACTCGTGGTAGACTCCTTC |  |
| *ACLY* | F：ACTACGCCAAGACGATCCTCTCC | 82 | *CASP3* | F：GTATTGAGACAGACAGTGGTGCTGAG | 96 |
|  | R：GCGATGCTGCCTCCGATGATG |  |  | R：AACCAGGTGCTGTAGAATATGCGTAC |  |
| *ACOX1* | F：GCGGTTGCTCTGGTGGATGC | 150 | *CD36* | F：TGCCAGTTGGAGACATGCTTATTGAG | 152 |
|  | R：GTGCTTGTAAGACTCATGGACCTCTG |  |  | R：TGTCACTTCATCTGGATTCTGCACATC |  |
| *ACSL5* | F：CACTGATCTGCATCCTGACCTTCG | 182 | *CEBPB* | F：GCACAGCGACGAGTACAAGATCC | 158 |
|  | R：CGTCTTGGCATCTGAGAAGTAGTAGC |  |  | R：GCGACAGTTGCTCCACCTTCTTC |  |
| *ACSM3* | F：ATTGGCATTCGAGTTCGACCTAACC | 135 | *CNBP* | F：GCTTCAAGTGCGGACGGTCTG | 88 |
|  | R：TCCATCCTCATCCATATAGCCTCTGTC |  |  | R：CCTCTGCCACGGCTTCTCATTC |  |
| *ADIPOR1* | F：GCAGGAGGAGGAGGAAGAGGTG | 157 | *CPT1a* | F：ACACCGTCAACAGGTACTTGGAATC | 84 |
|  | R：GTAGTCGTTGTCCTTCAGCCAGTC |  |  | R：AATCTTGTGCCAGAGCCGTCATC |  |
| *ADIPOR2* | F：GAGGAGCGTGAGTGCGATGATG | 196 | *CPT2* | F：AGACAGAGGCTGGTGAGTGGTG | 84 |
|  | R：GTCCATGCAGGAGGAAGTCGTTATC |  |  | R：GAAGTCATCTAGGCAGAGACAGAACAC |  |
| *AKT1* | F：AGGACCTGGAGCAGCGTGAG | 108 | *CYP2E1* | F：GAGGCTGATGAGTCTGTTCAACGAG | 101 |
|  | R：GCAGGCAGCGGATGATGAAGG |  |  | R：TTCCAGGCAGGTACTGTAGGTAGTC |  |
| *APOA1* | F：GCTGACCTTGGCTGTGCTCTTC | 137 | *CYP7A1* | F：GGCTTGAGACTCCAGAAGTTCAGAG | 119 |
|  | R：CTCTGCCACTATCCTTGATTGCTTCC |  |  | R：AGGACTGCGAGGAGTGACTTGG |  |
| *DGAT2* | F：ACACAACTTACTGACCAGCAGGAAC | 198 | *IFNG* | F：GCAGCTCTGAGAAACTGGAGG | 80 |
|  | R：ATGCCTCCAGACATCAGGTACTCC |  |  | R：TATGGCTTTGCGCTGGATCTG |  |
| *FABP1* | F：TCTCCGGCAAGTACCAAGTCCAG | 161 | *IGF1* | F：ATCCTCCTCGCATCTCTTCTATCTGG | 119 |
|  | R：GGAGCCAGCGGTGATGATGAAC |  |  | R：CGCACACGAACTGGAGAGCATC |  |
| *FABP3* | F：CGATGAGACCACAGCAGATGACAG | 100 | *IGFBP1* | F：TTGATGGCCGAGTCCAGTGAGG | 172 |
|  | R：TGATGTCTCTTGTCCATTCCACTTCTG |  |  | R：TGTCTCCTGCCTTCTGCTGCTC |  |
| *FABP5* | F：CGCATTGGTTCAACATCAGGAATGG | 105 | *IL10* | F：CACTACTCTGTTGCCTGGTCTTCC | 181 |
|  | R：CAGGTAACGTTGTTCATGACGCATAC |  |  | R：AGTAAGCTGTGCAGTTGGTCCTTC |  |
| *FAS* | F：ATTACTGAAGCGAACTCCTGCCAAG | 163 | *IL1B* | F：TGATGGCTTACTACAGTGACGAGAATG | 169 |
|  | R：GGCTCTTGTCTGTGTACTCGTTCC |  |  | R：ACCGACACCACCTGCCTGAAG |  |
| *FASN* | F：CTGAGTCGGAGAACCTGGAGGAG | 83 | *IL6* | F：CACTGACCTGCTGGAGAAGATGC | 115 |
|  | R：CTTCCACCGCCTGTCATCATCTG |  |  | R：CCGAATAGCTCTCAGGCTGAACTG |  |
| *FOXA2* | F：TCTACCAGTGGATCATGGACCTCTTC | 84 | *INSR* | F：AGTCAGCCAGCCTGCGAGAG | 139 |
|  | R：AGGACAGCGAGTGGCGGATG |  |  | R：GCCATCAACTCCATCACCACCAG |  |
| *G6PC* | F：AGTCTTGTCAGGCATTGCGGTTG | 104 | *IRS1* | F：CGTGCGTGCCTCCAGTGATG | 139 |
|  | R：AACTGAACAGGAAGCAGGTGATGAG |  |  | R：GAAGGCGAGCAGCGAGAAGAAG |  |
| *GCK* | F：GAAGACCACGCACCAGATGTACTC | 110 | *LDLR* | F：AGCCAAGCGGACACGGAGAG | 180 |
|  | R：GATGCTTATCCAGGAAGTCAGAGATGC |  |  | R：GGTTGTCGAAGTTGATGCTGTTGATG |  |
| *GSK3B* | F：CAACTTCACCACTCAAGAACTGTCAAG | 138 | *LEPR* | F：GTGCCACCATCGCTATGCTGAG | 148 |
|  | R：CCACGGTCTCCAGCATTAGCATC |  |  | R：GAACAGTACAGGCTGCTCCTATGATAC |  |
| *HMGCR* | F：TGGTTCTTGTTCACGCTCACAGTC | 124 | *LPL* | F：GTCGCCGCAGACAGGATTACAG | 171 |
|  | R：ACACTCGGTTCAATTCTCTTGGACAC |  |  | R：TGAAGTGACAGTTAGCCACAGATTCG |  |
| *HNF4A* | F：TTGCCAACACGATGCCTGCTC | 185 | *MAPK1* | F：AACCTTCCAACCTGCTGCTCAAC | 142 |
|  | R：CTAGATGACTTCCTGCTTGGTGATGG |  |  | R：CAGATGTCGATGGACTTGGTGTAGC |  |
| *MAPK8* | F：GAATGTCCTACCTGCTCTATCAGATGC | 184 | *PPA1* | F：GCCGCGCTGGTCGAATGC | 142 |
|  | R：GCGAGTCACTACGTAAGGTGTCATC |  |  | R：GATGGCACCGTAGTTCCAGATGTATC |  |
| *MEN1* | F：GATGGAGGTGGCATTTATGG | 188 | *PPARa* | F：ATCAGATGGCTCCGTTATTACAGACAC | 131 |
|  | R：GATGTGCTCATCCCGGTAGT |  |  | R：CGCAGATCCTACACTCGATGTTCAG |  |
| *mTOR* | F：CGTCTCGCTTGTACTTTGGG | 87 | *PPARD* | F：GGCTTCCACTACGGCGTTCAC | 129 |
|  | R：GCTGCTTGGAGATTCGTCTG |  |  | R：GCACTTGTTGCGGTTCTTCTTCTG |  |
| *NDUFB6* | F：AGGCGATGGCTGAAAGATCA | 88 | *PPARG* | F：CTGTGAAGTTCAACGCACTGGAATTAG | 133 |
|  | R：ATTCCAGAATCGCTCCACAGG |  |  | R：TGCAGCAGATTGTCTTGTATGTCCTC |  |
| *NFKB1* | F：TGTGGTGGAGGACTTGCTGAGG | 189 | *PPARGC1A* | F：CATTGTTCGATGTGTCGCCTTCTTG | 176 |
|  | R：ATGGCTACATGGATGGCATTCAGAC |  |  | R：GGACCTTGATCTTGACCTGGAATATGG |  |
| *NR1H2* | F：GACGCTACAACCACGAGACTGAG | 195 | *PRKAA1* | F：ACTGCTACGCCACAGAGATCGG | 136 |
|  | R：GGTCGGCTGAGAAGATGTTGATGG |  |  | R：CAGGAGAAGAGTCAAGTGAGGTTACAG |  |
| *NR1H3* | F：GGTTCTTCCGCCGCAGTGTC | 200 | *PTPN1* | F：CTGACACCTGCCTCTTGCTGATG | 129 |
|  | R：CTGTTCCTCCTCTTGCCGCTTC |  |  | R：AGGAGAAGCGGAGCTGGTCTG |  |
| *NR1H4* | F：TCTGTGGAGACCGAGCATCTGG | 157 | *RBP4* | F：ACTCCTGCCGCCTCCTGAAC | 185 |
|  | R：TCTTGGCACTTCCTTCGCATGTAC |  |  | R：TTCTGACTTGCCATCACAGTAACCG |  |
| *PDK4* | F：GCCAGGATACGGAACTGATGCTATC | 117 | *RXRa* | F：GGCGTCCTTCACCAAGCACATC | 161 |
|  | R：GCCTCGCTGCTCGTCTGATAATG |  |  | R：TGTCAATCAGGCAGTCCTTGTTGTC |  |
| *PIK3CA* | F：TCCTGATCTTCCTCGTGCTGCTC | 93 | *SERPINE1* | F：GAGAGCCAGGTTCATCGTCAACG | 199 |
|  | R：CAGGCCAATGGACAGTGTTCCTC |  |  | R：GGTGCTGCCATCGGACTTGTG |  |
| *PIK3R1* | F：TGAGAAGACTTGGAATGTTGGAAGCAG | 129 | *SLC27A5* | F：GCTTGTCCTTGGAGTCCTCAGTTG | 104 |
|  | R：ACCACCACAGAGCAGGCGTAG |  |  | R：CACCATGCTGCCGACAGTCATC |  |
| *PKLR* | F：GTGGAGCATGGCGTGGACATC | 180 | *SLC2A1* | F：CGGCTCTGGCATCGTCAACAC | 180 |
|  | R：CACCATAATACCGTCGCTCACCTC |  |  | R：GCCACAATGCTCAGGTAGGACATC |  |
| *SLC2A2* | F：CACTGCTGTGCTGAGTTCCTTCC | 150 | *SREBF2* | F：GTCCTCACCTTCCTCTGCCTCTC | 188 |
|  | R：TCTGTACTGTTGAGAGCGTAGTTGTTG |  |  | R：ATCATGCCATTCACCAGCCACAG |  |
| *SLC2A4* | F：TCATTCTTGGACGGTTCTTCATTGGAG | 134 | *STAT3* | F：GAGAAGGACATCAGCGGCAAGAC | 143 |
|  | R：AGTGACGATGGCTAGTTGATTGAGTG |  |  | R：TAGACCAGTGGAGACACCAGGATATTG |  |
| *SLC2A4* | F：TCATTCTTGGACGGTTCTTCATTGGAG | 134 | *TNF* | F：CTGGCGGAGGAGGTGCTCTC | 85 |
|  | R：AGTGACGATGGCTAGTTGATTGAGTG |  |  | R：GGAGGAAGGAGAAGAGGCTGAGG |  |
| *SOCS3* | F：GCCTCAAGACCTTCAGCTCCAAG | 120 | *XBP1* | F：ATGGACTCTGATGGCATTGACTCTTC | 99 |
|  | R：TCAGCAGCAAGTTCGCTTCGC |  |  | R：GGAAGGACATCTGAGGAACATGACTG |  |
| *SREBF1* | F：CATCGACTACATCCGCTTCCTTCAG | 112 |  |  |  |
|  | R：AGCCGACACCAGGTCCTTCAG |  |  |  |  |

**Table S3. The primers information used for Menin-ChIP and/or SIRT1-ChIP assays in the mouse hepatocytes, designed in the promoter region of PPARγ targeted genes.**

| Gene | Primers | Primer sequences (5’→3’) | Product size | Starting position | End position |
| --- | --- | --- | --- | --- | --- |
| *Fabp3* P1 | Forward | GCCTACACGAGCATACACAA | 208 | -1100 | -1308 |
|  | Reverse | GTCCTGTTGGTGCCATCTA |  |  |  |
| *Fabp3* P2 | Forward | GGTGTCCAAGGCAACTCTT | 316 | -801 | -1117 |
|  | Reverse | TGTATGCTCGTGTAGGCGT |  |  |  |
| *Fabp3* P3 | Forward | CTACAACCATCAATAGTCGGG | 323 | -493 | -816 |
|  | Reverse | TGACCTTGCTTCCCTCAT |  |  |  |
| *Fabp3*P4 | Forward | AGAGAGTATCTTGCCTTGGC | 339 | -203 | -542 |
|  | Reverse | AGACCCGACTATTGATGGTT |  |  |  |
| *Fabp3* P5 | Forward | GGATGCTCTACTTGGGTTGC | 167 | -1228 | -1395 |
|  | Reverse | TCGCTCCCGAAATAGGAA |  |  |  |
| *Fabp4* P1 | Forward | GGGTCTTATCCAGTAGGAAGC | 255 | -633 | -888 |
|  | Reverse | CAGGGTGTTAGAGAGAGATGG |  |  |  |
| *Fabp4* P2 | Forward | CCATCTCTCTCTAACACCCTG | 300 | -868 | -1208 |
|  | Reverse | CTTCCCATTCGTAAATAGCC |  |  |  |
| *Fabp4* P3 | Forward | CGAATGGGAAGAATAAGGC | 273 | -1198 | -1471 |
|  | Reverse | GGCTTTGTTTGGTTTGGG |  |  |  |
| *Fabp4* P4 | Forward | CCACAATGAGGCAAATCC | 245 | -1746 | -1991 |
|  | Reverse | GACTGTAGGAGTGACCAATGG |  |  |  |
| *Fabp5* P1 | Forward | GAGTCTTCCATCCTGGGTT | 334 | -1008 | -1342 |
|  | Reverse | AGCCACCTGTGATAGTCCA |  |  |  |
| *Fabp5* P2 | Forward | GGACTATCACAGGTGGCTTAC | 330 | -701 | -1031 |
|  | Reverse | ATCCGCTGACAGTTAGATGC |  |  |  |
| *Fabp5* P3 | Forward | CTAACTGTCAGCGGATTGTCC | 327 | -328 | -655 |
|  | Reverse | CCTCTTGGTTGTCTGTCTGTG |  |  |  |
| *Fabp5* P4 | Forward | AGAGACTGCTGCCAGGATT | 167 | -47 | -214 |
|  | Reverse | TGTGCCTCCCTTAGTGACA |  |  |  |

**Table S4. The primers information used for Menin-ChIP assays in the liver tissues of dairy cows, designed in the promoter region of PPARγ target genes.**

| Gene | Primers | Primer sequences(5’→3’) | Product size (bp) | Starting position | End position |
| --- | --- | --- | --- | --- | --- |
| *FABP3 P1* | Forward | ACCCACACAGTTAGCACCC | 329 | -1534 | -1863 |
|  | Reverse | ACAGGTCCACTTGCCAGA |  |  |  |
| *FABP3* P2 | Forward | TTCTGGCAAGTGGACCTG | 300 | -1552 | -1252 |
|  | Reverse | CTGGAGAGACAACTGGGAAC |  |  |  |
| *FABP3* P3 | Forward | GGAAGGGAGGCAGATGTT | 214 | -1252 | -1466 |
|  | Reverse | CTGGAGAGACAACTGGGAAC |  |  |  |
| *FABP3* P4 | Forward | TCGGAAGGAGAGTGAGGA | 345 | -508 | -853 |
|  | Reverse | GCAGGAAATAAGGAGACGG |  |  |  |
| *FABP4* P1 | Forward | GCTCTTATGGGAAGCCAA | 206 | -806 | -1012 |
|  | Reverse | AGAATACTGGAGTGGGTTGC |  |  |  |
| *FABP4* P2 | Forward | AGTCCATAGGGTGGCAAA | 274 | -1212 | -148 |
|  | Reverse | GCTAAGTCGCTTCAGTTGTG |  |  |  |
| *FABP4* P3 | Forward | CAGACACACACACACACACACT | 324 | -1502 | -1826 |
|  | Reverse | CCCTCAGCCTTCTTCACAG |  |  |  |
| *FABP5* P1 | Forward | CCCTGCCTCATTTCTCTTTC | 216 | -354 | -638 |
|  | Reverse | TTGTGCTCCTGGGTTGCT |  |  |  |
| *FABP5* P2 | Forward | CGACTCACTCCCACAGCAA | 291 | -618 | -909 |
|  | Reverse | TCCCAGGCAGTTCGGTTT |  |  |  |
| *FABP5* P3 | Forward | TGGAAACCGAACTGCCTG | 284 | -878 | -1094 |
|  | Reverse | CGTGACACCCTCAAAGTATCG |  |  |  |
