# Supplementary material for: Menin orchestrates hepatic glucose and fatty acid uptake via deploying the cellular translocation of SIRT1 and PPARγ
Source: Cell Biosci. 2023 Sep 22;13:175. doi: 10.1186/s13578-023-01119-y (PMC10517496; doi:10.1186/s13578-023-01119-y)
Supplement: Supplementary file 2 — Additional file 2: Figure S1. Menin was abundently expressed in metabolism-associated tissues in cattle. A. Real-time quantitative PCR results of MEN1 gene in different tissues of calves. The same data with the same letter is not significantly different (P > 0.05), and the different letters are the same (P < 0.05). B. Western blot results of Menin protein in different tissues of calves. The same data with the same letter is not significant (P > 0.05), and the different letters with the top is significant (P < 0.05). C. Immunohistochemical staining of Menin in different tissues of calves. The letters A, B, C, D, E and F at lower right corners represents the immunity histochemical staining results from liver, kidney, pancreas, testis, duodenal mucosa and duodenal muscle layer, respectively. Figure S2. Liver-specific fatty acid transporter Fabp1 in hepatocytes was found inhibited upon Menin higher- and/or lower expression. The protein expression of Fabp1 was shown to be suppressed in both conditions of Menin over-expression by transfecting Men1 cds clone (mMen1) and Menin low-expression by transfecting Men1 specific siRNA (si-Men1), compared with their negative controls (Vector and control) transfected cells. Figure S3. Menin knockdown induced inflammation in hepatocytes. The expresssion of inflammation promoting factors Il1α and Il1β were detected to be enhanced in Menin-specific siRNA (si-Men1) treated mouse hepatocytes (NCTC-1469) at 24 h after transfection, compared with that of nonspecific negative control siRNA (Ctrl) treated cells. Figure S4. Optimization of treatment conditions for fatty liver cell model induction using sodium oleate (OA). A. The viability of NCTC-1469 cells was measured under different concentrations of OA, indicating that 0.25 mM OA was the optimum condition; B. Upon treatment of NCTC-1469 cells with 0.25 mM OA for 24 h, 48 h and 72 h, the accumulate TAG (triglyceride) in cells was significantly increased, with lipid droplets appearance. Figu [file 13578_2023_1119_MOESM2_ESM.docx]

**Menin orchestrates hepatic metabolic homeostasis via deploying glucose uptake and fatty acid uptake**

Tingjun Liu^1, 2,#^, Ranran Li^1#^, Lili Sun^1^, Zhongjin Xu^1^, Shengxuan Wang^1^, Jingxuan Zhou^1,2^, Xuanning Wu^1, 2^, Kerong Shi^1, 2, *^


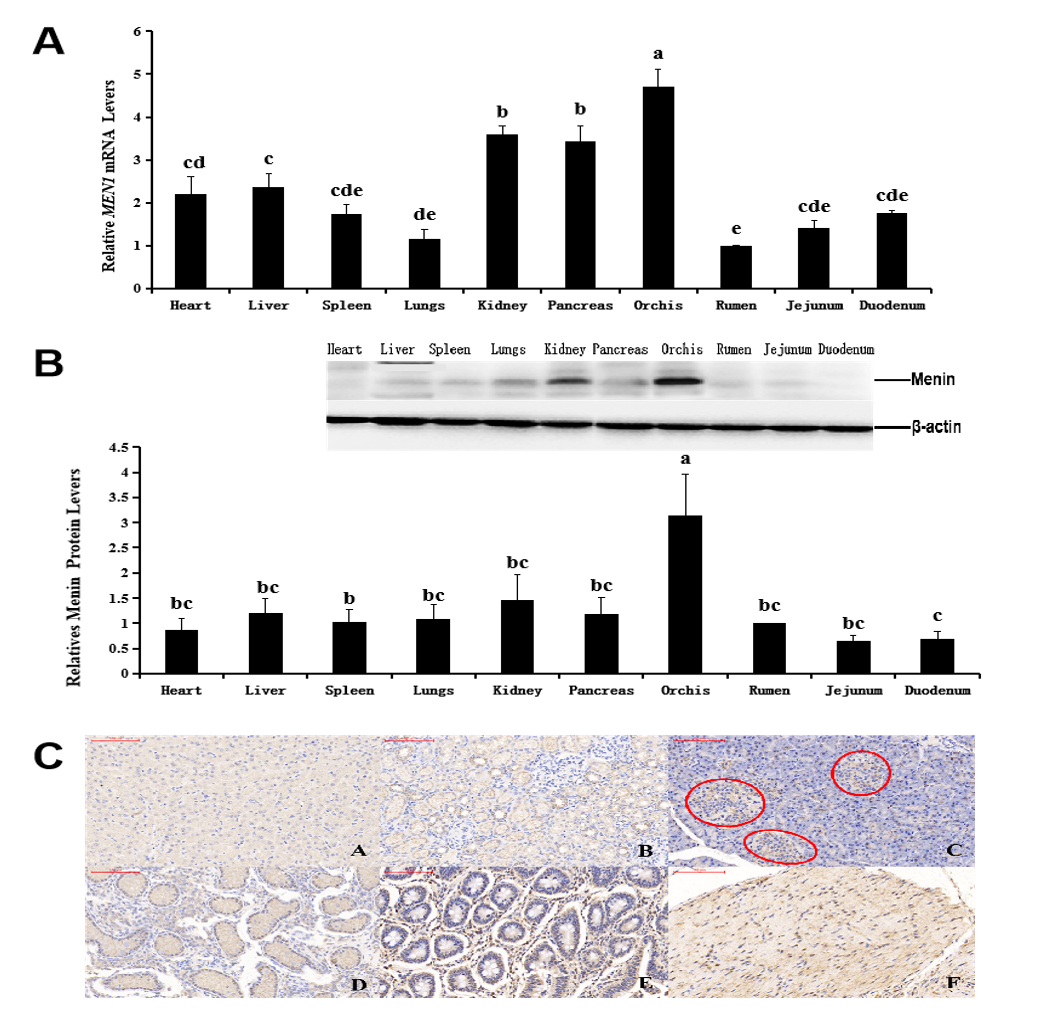


**Fig S 1. Menin was abundently expressed in metabolism-associated tissues in cattle.**  **A.** Real-time quantitative PCR results of MEN1 gene in different tissues of calves. The same data with the same letter is not significantly different (P> 0.05), and the different letters are the same (P <0.05). **B.** Western blot results of Menin protein in different tissues of calves. The same data with the same letter is not significant (P> 0.05), and the different letters with the top is significant (P <0.05). **C.** Immunohistochemical staining of Menin in different tissues of calves. The letters A, B, C, D, E and F at lower right corners represents the immunity histochemical staining results from liver, kidney, pancreas, testis, duodenal mucosa and duodenal muscle layer, respectively.


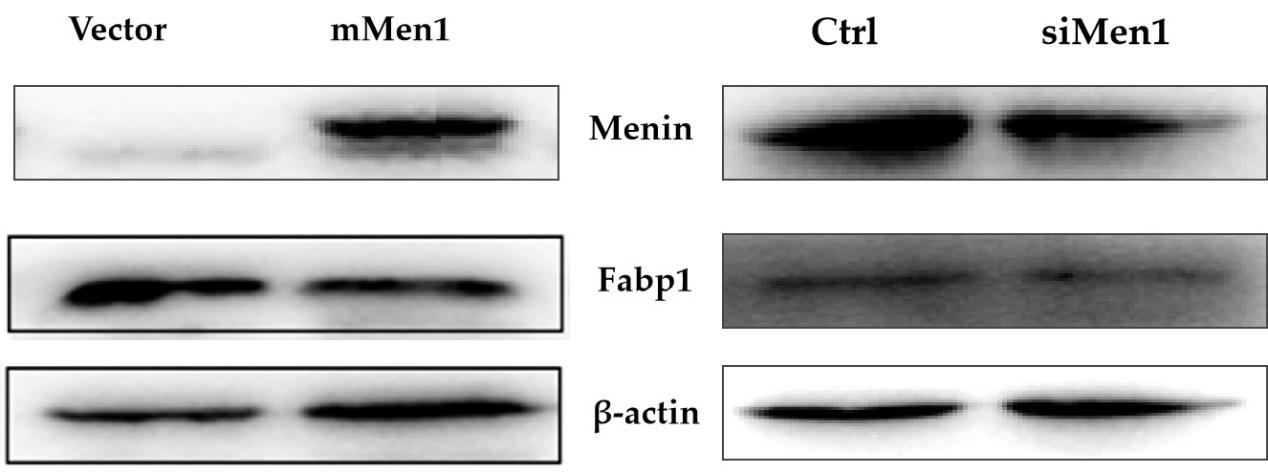


**Fig S2. Liver-specific fatty acid transporter Fabp1 in hepatocytes was found inhibited upon Menin higher- and/or lower expression.** The protein expression of Fabp1 was shown to be suppressed in both conditions of Menin over-expression by transfecting Men1 cds clone (mMen1) and Menin low-expression by transfecting Men1 specific siRNA (si-Men1), compared with their negative controls (Vector and control) transfected cells.


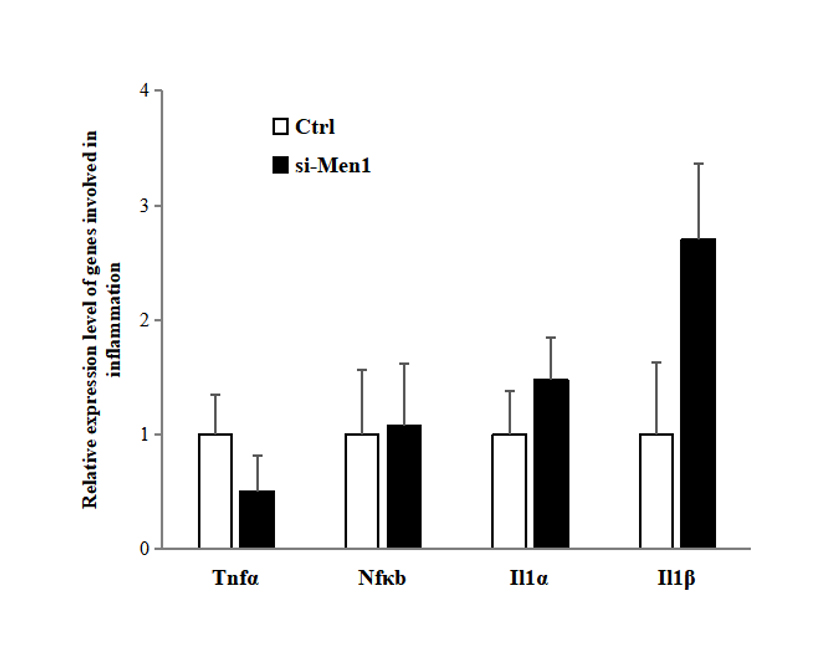


**Fig S3. Menin knockdown induced inflammation in hepatocytes.** The expresssion of inflammation promoting factors Il1α and Il1β were detected to be enhanced in Menin-specific siRNA (si-Men1) treated mouse hepatocytes (NCTC-1469) at 24h after transfection, compared with that of nonspecific negative control siRNA (Ctrl) treated cells.


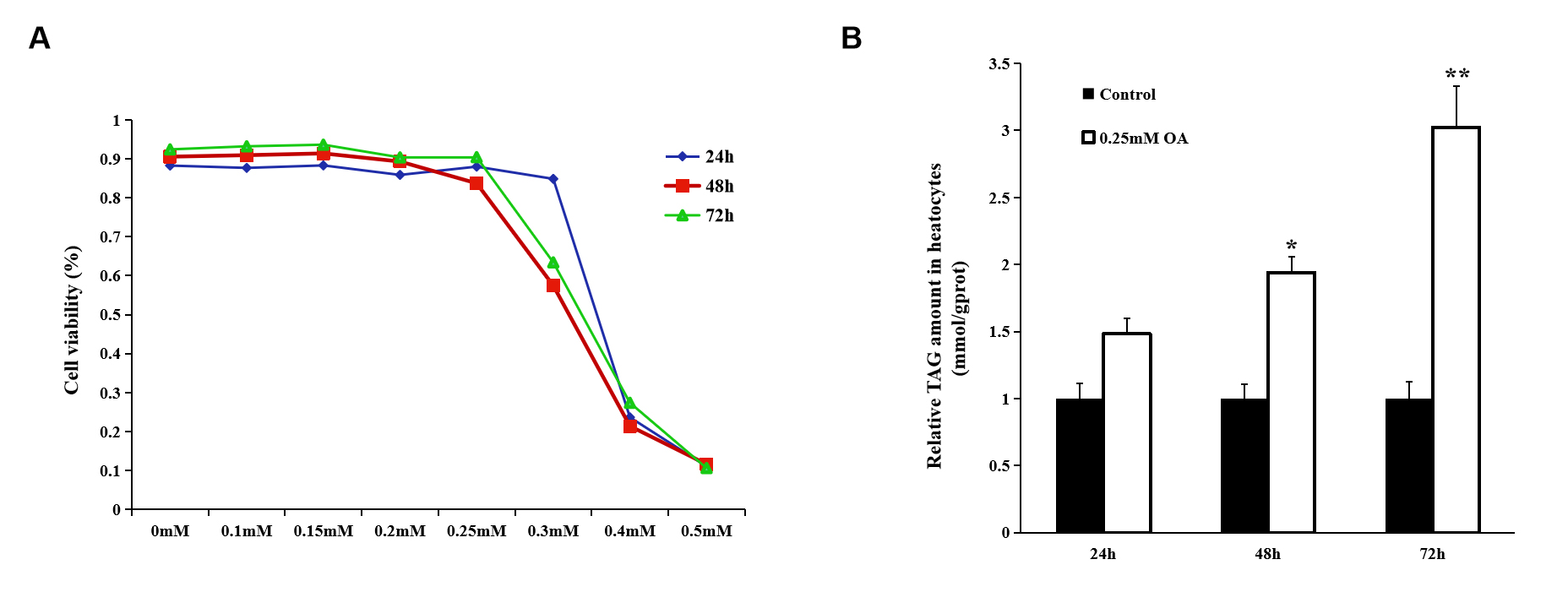


**Fig S4. Optimization of treatment conditions for fatty liver cell model induction using sodium oleate (OA).**  **A.** The viability of NCTC-1469 cells was measured under different concentrations of OA, indicating that 0.25mM OA was the optimum condition; **B.** Upon treatment of NCTC-1469 cells with 0.25 mM OA for 24h, 48h and 72h, the accumulate TAG (triglyceride) in cells was significantly increased, with lipid droplets appearance.


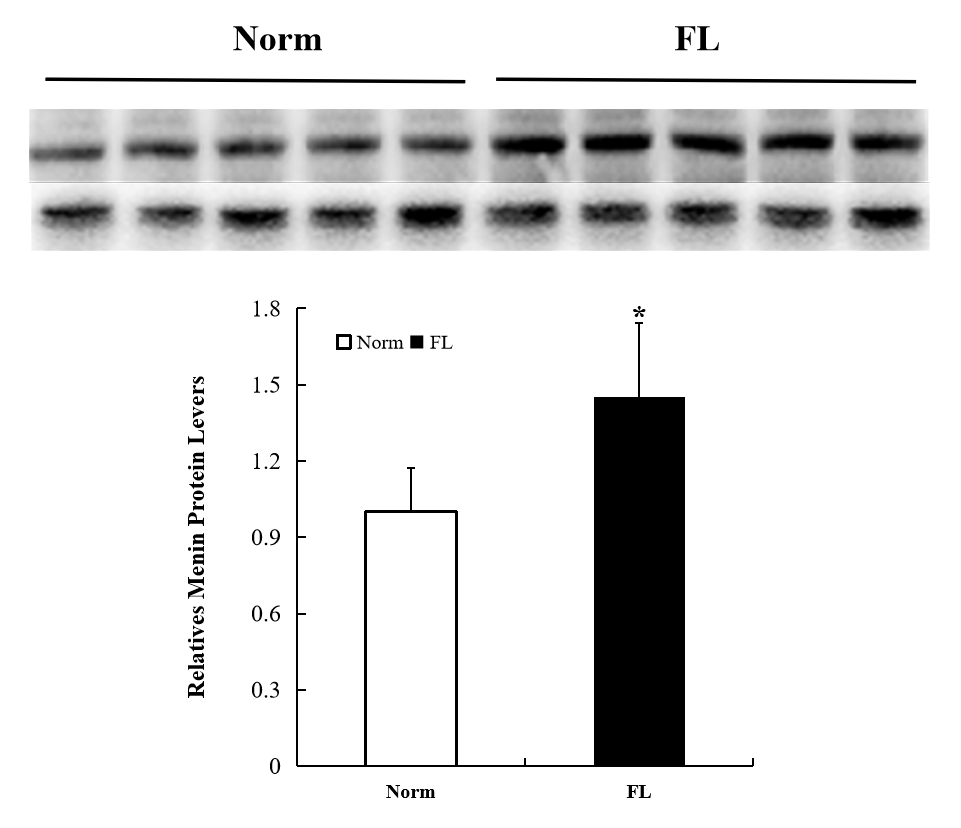


**Fig S5. Menin expression was elevated in fatty liver tissues of dairy cows, compared with that in normal livers. A.** WB results of Menin protein expression in biopsied normal (Norm) and fatty liver (FL) tissues from different prenatal dairy cows. **B.** Quantitative results of WB results indicated Menin expression was elevated in biopsied fatty liver tissues.


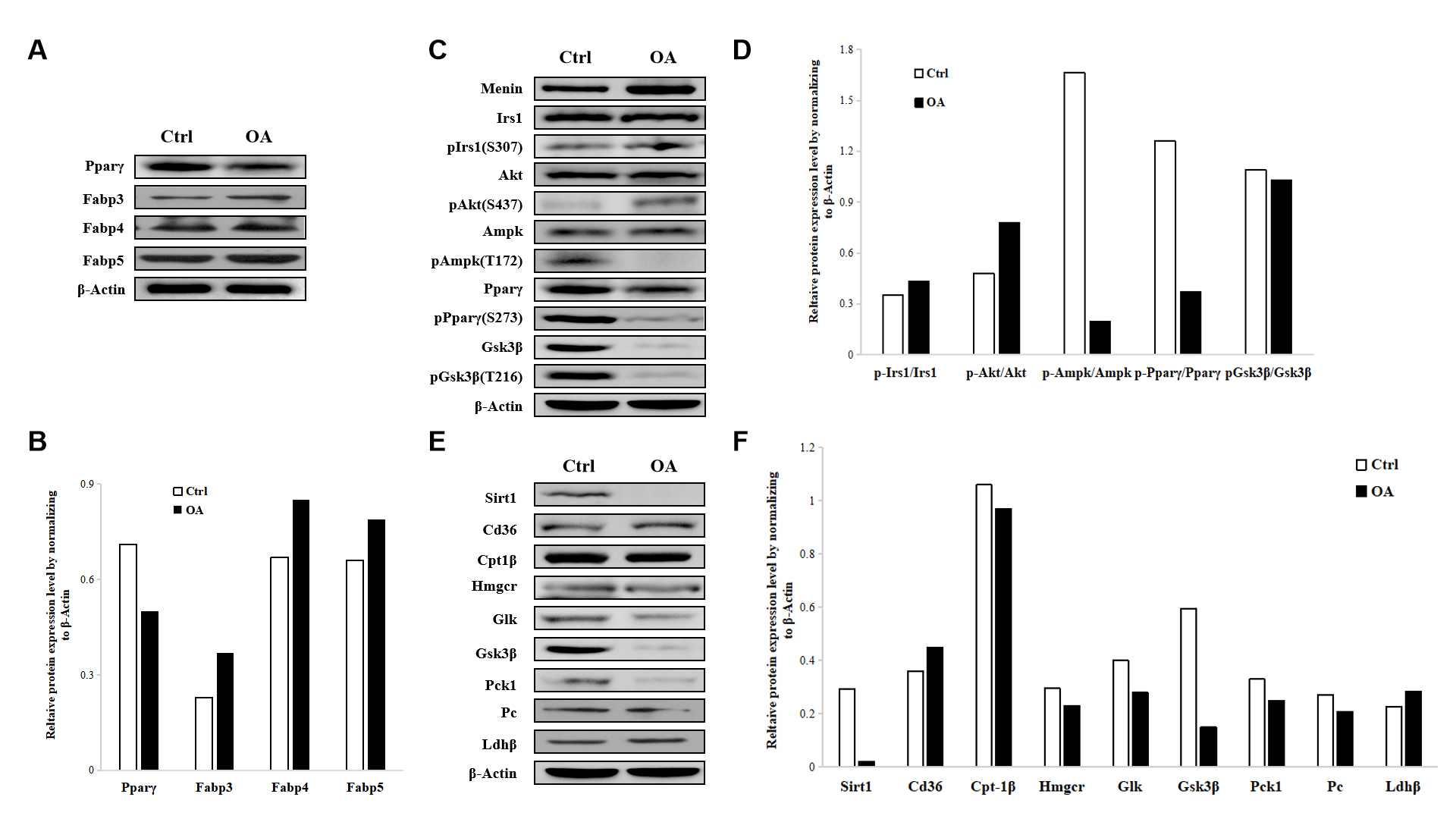


**Fig S6. OA induction, producing a fatty cell model, activates insuling pathway and Akt, but inhibites the activity of Ampk, Pparγ and Gsk3β, enhancing fatty acid uptake and adopogenesis, but suppressing glucose uptake and glugluconeogenesis. A.** Representative WB results of key mediator factors insulin receptor (activated Irs1 being phosphorylated at S307, and its total protein), Akt (activated Akt being phosphorylated at S437, and its total protein), Ampk (activated Ampk being phosphorylated at T172, and its total protein), Pparγ (activated Pparγ being phosphorylated at S273, and its total protein) and Gsk3β (activated Gsk3β being phosphorylated at T216, and its total protein), upon OA induction in hepatocytes. **B.** Quantitative results of WB results obtained from OA treated whole cell extracts, indicating that enhanced activity of Irs1, Akt, but inhibited activity of Ampk and Pparγ and/or Gsk3β. **C.** Representative WB results of down-stream factors involved in Ppar signaling pathway upon OA treatment in hepatocytes, showing increased fatty acid receptor Cd36, but inhibited rate-limit enzyme of glucose uptake Gk and gluconeogenesis Pck, Gsk3β. **D.** Quantitative results of WB results from OA treated whole cell extracts, indicating that OA induction facilitates fatty acid uptake (Cd36) and lipid synthesis, whileas suppresses gucose uptake (Gk) and glugluconeogenesis (Pck).
